# Epigenetic drug screening defines a PRMT5 inhibitor–sensitive pancreatic cancer subtype

**DOI:** 10.1172/jci.insight.151353

**Published:** 2022-05-23

**Authors:** Felix Orben, Katharina Lankes, Christian Schneeweis, Zonera Hassan, Hannah Jakubowsky, Lukas Krauß, Fabio Boniolo, Carolin Schneider, Arlett Schäfer, Janine Murr, Christoph Schlag, Bo Kong, Rupert Öllinger, Chengdong Wang, Georg Beyer, Ujjwal M. Mahajan, Yonggan Xue, Julia Mayerle, Roland M. Schmid, Bernhard Kuster, Roland Rad, Christian J. Braun, Matthias Wirth, Maximilian Reichert, Dieter Saur, Günter Schneider

**Affiliations:** 1 Medical Clinic and Polyclinic II, Klinikum rechts der Isar and; 2 Institute for Translational Cancer Research and Experimental Cancer Therapy, Technical University Munich (TUM), Munich, Germany.; 3 University Medical Center Göttingen, Department of General, Visceral and Pediatric Surgery, Göttingen, Germany.; 4 Department of Surgery, Klinikum rechts der Isar, TUM, Munich, Germany.; 5 Department of General Surgery, University of Ulm, Ulm, Germany.; 6 Institute of Molecular Oncology and Functional Genomics, TUM School of Medicine and; 7 Chair of Proteomics and Bioanalytics, TUM School of Life Sciences, TUM, Freising, Germany.; 8 Department of Pediatric Surgery, Xinhua Hospital, School of Medicine, Shanghai Jiaotong University, Shanghai, China.; 9 Department of Surgery, Children’s Hospital of Soochow University, Suzhou, China.; 10 Department of Medicine II, LMU University Hospital, Ludwig-Maximilians-Universität München (LMU Munich), Munich, Germany.; 11 Bavarian Cancer Research Center (BZKF), Munich, Germany.; 12 German Cancer Research Center (DKFZ) and German Cancer Consortium (DKTK), Heidelberg, Germany.; 13 Bavarian Center for Biomolecular Mass Spectrometry (BayBioMS), TUM, Freising, Germany.; 14 Department of Pediatrics, Dr. von Hauner Children’s Hospital, University Hospital, LMU Munich, Munich, Germany.; 15 Department of Hematology, Oncology and Tumor Immunology, Campus Benjamin Franklin, Charité – Universitätsmedizin Berlin, Berlin, Germany.; 16 Center for Protein Assemblies (CPA), TUM, Garching, Germany.; 17 Translational Pancreatic Research Cancer Center, Medical Clinic and Polyclinic II, Klinikum rechts der Isar, TUM, Munich, Germany.

**Keywords:** Cell Biology, Oncology, Cancer, Pharmacology

## Abstract

Systemic therapies for pancreatic ductal adenocarcinoma (PDAC) remain unsatisfactory. Clinical prognosis is particularly poor for tumor subtypes with activating aberrations in the MYC pathway, creating an urgent need for novel therapeutic targets. To unbiasedly find MYC-associated epigenetic dependencies, we conducted a drug screen in pancreatic cancer cell lines. Here, we found that protein arginine *N*-methyltransferase 5 (PRMT5) inhibitors triggered an MYC-associated dependency. In human and murine PDACs, a robust connection of MYC and PRMT5 was detected. By the use of gain- and loss-of-function models, we confirmed the increased efficacy of PRMT5 inhibitors in MYC-deregulated PDACs. Although inhibition of PRMT5 was inducing DNA damage and arresting PDAC cells in the G2/M phase of the cell cycle, apoptotic cell death was executed predominantly in cells with high MYC expression. Experiments in primary patient-derived PDAC models demonstrated the existence of a highly PRMT5 inhibitor–sensitive subtype. Our work suggests developing PRMT5 inhibitor–based therapies for PDAC.

## Graphical abstract

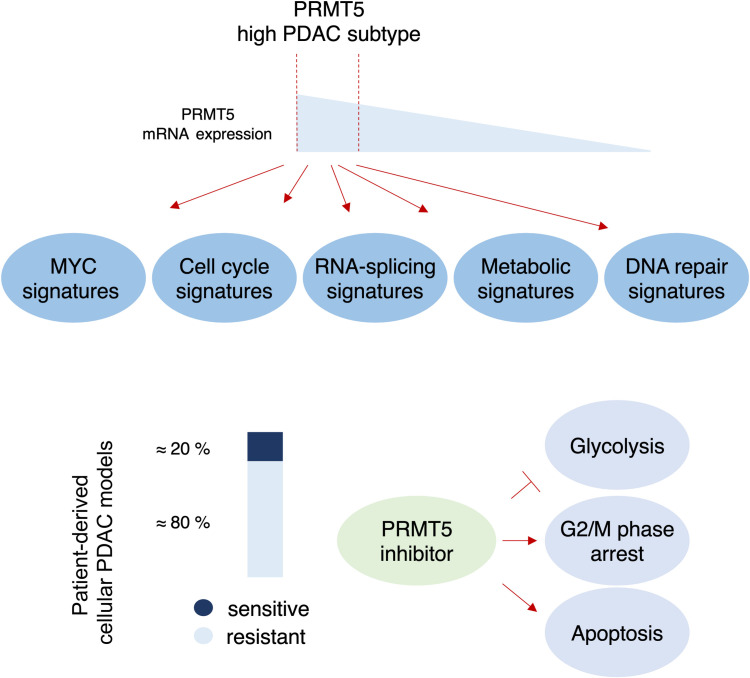

## Introduction

The specific tumor context is an important and relevant aspect for the definition of therapeutic targets and the development of novel therapies (1). This conclusion is underscored by genome-scale CRISPR/Cas9 dropout screens. A functional screen across 30 tumor entities allowed prioritization of targets and demonstrated that a median of 88 priority targets per tumor entity exists. Importantly, these tumor entity–specific targets were defined in 56% of cases in only 1 cancer type (2). Such data demonstrate the need to conduct screens for therapeutic vulnerabilities in a context-dependent manner.

The incidence and mortality of PDAC are increasing: the disease will be the second leading cause of cancer-related death soon, and the current 5-year survival rate is only 10% (3, 4). In approximately 80% to 85% of patients with locally advanced or disseminated disease, a chemotherapeutic regimen with folinic acid, 5-fluorouracil, irinotecan, and oxaliplatin (FOLFIRINOX) or nab-paclitaxel combined with gemcitabine are the standard of care (5). However, such therapies result in overall response rates of only 20% to 30%, and adverse events affecting quality of life have to be considered (5). Therefore, novel and active therapies are needed.

Deregulation of the myelocytomatosis oncogene MYC is a frequent event in cancer (6), and the oncogene is an important driver of pancreatic ductal adenocarcinoma (PDAC; refs. 7–9). Amplification of MYC is associated with worse survival of patients with PDAC (10) and a driver of metastatic heterogeneity (11). MYC binds as a dimer with MYC-associated protein X (MAX) to DNA and regulates genes involved in RNA metabolism, ribosome biogenesis, metabolism, nucleotide synthesis, mitochondrial biogenesis, and the cell cycle (6). Although meaningful progress has been made (12), MYC remains a challenge from the therapeutic view. One road to target cancers with oncogenic MYC activity is to exploit cellular vulnerabilities associated with the transcription factor. Unbiased genetic screening experiments demonstrated the existence of such synthetic lethal interactions of MYC and its paralogues (13–16). Such interactions also occur in the context of PDAC and can be targeted, for example, by BET inhibitors (17), SUMOylation inhibitors (18, 19), or perturbants of protein homeostasis (20).

MYC interacts directly or indirectly with a broad repertoire of epigenetic modifiers and thereby influences the epigenome of cancer cells (21). Considering the relevance of epigenetic therapies in PDAC (22), we set up experiments to define epigenetic MYC-associated vulnerabilities using an unbiased pharmacological screening experiment. Here, we observed inhibitors of the protein arginine *N*-methyltransferase 5 (PRMT5) as screening hits. We detected a robust connection of PRMT5 to MYC in PDACs across species and gain- and loss-of-function models, demonstrating the tuning of PRMT5 inhibitor (hereafter, PRMT5i) sensitivity by MYC. Consistently, cell death was preferentially induced in models with higher MYC expression. Furthermore, a highly PRMT5i-sensitive PDAC subtype was defined in human primary patient-derived PDAC models, demonstrating the therapeutic potential.

## Results

### Unbiased epigenetic drug screen in PDAC cells with different MYC activation status.

**Figure 1:**
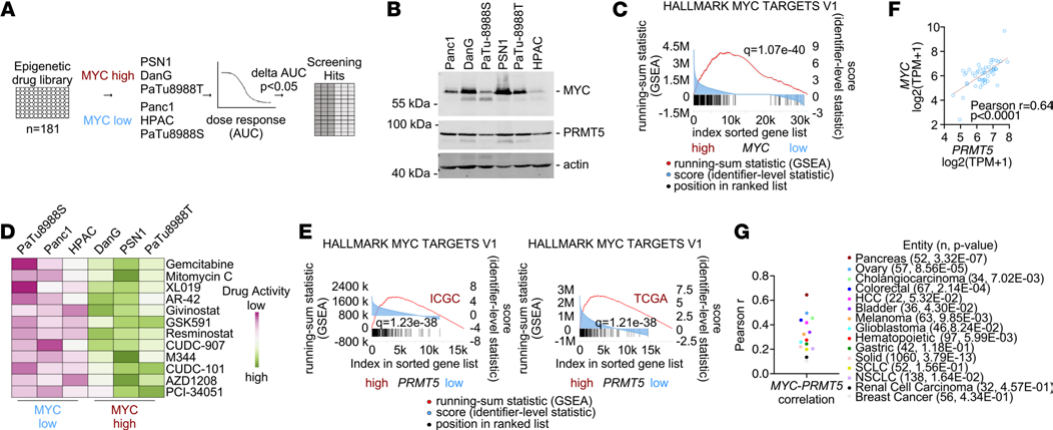
Epigenetic drug screening in human PDAC cells with diverse MYC activity. (**A**) Strategy for drug screening experiments using a library of *n* = 181 epigenetic drugs. Cells were treated for 72 hours with 7 concentrations (maximum 10 μM) of each compound. Hits were determined as difference in the mean area under the dose-response curve (AUC) between MYC-high and MYC-low cell lines with *P* < 0.05. Screening was conducted as 1 biological replicate in technical triplicates. (**B**) MYC and PRMT5 protein expression of the 6 indicated cell lines determined by Western blotting. β-Actin: loading control. One representative experiment out of 3 is shown. (**C**) GSEA of the MYC-high and MYC-low cell lines depicted in **A** was performed using the GeneTrail3 web tool. Illustrated is the enrichment plot of the HALLMARK signature MYC TARGETS V1, including the *q* value. (**D**) Hits of the drug screening depicted as a variance scaled heatmap using AUC values as an input. (**E**) GSEA of RNA expression data sets with high PRMT5 (expression > 75th percentile) versus low PRMT5 (expression < 75th percentile) mRNA expression with curated TCGA (*n* = 150) and ICGC (*n* = 81) data sets. Depicted are the HALLMARK signatures for MYC TARGETS V1, including *q* values. (**F**) Depicted is the Pearson correlation coefficient and the linear regression (in red) between *MYC* and *PRMT5* mRNA expression in conventional human PDAC cell lines. Data were directly retrieved from the DepMap portal (*n* = 52). (**G**) Pearson’s correlation coefficient between *MYC* and *PRMT5* mRNA expression in the depicted tumor entities. Data were directly retrieved from the DepMap portal; *P* value is indicated.

To unbiasedly define MYC-associated epigenetic vulnerabilities, we used human PDAC cell lines with either high or low MYC activation status in combination with an epigenetic drug library containing 181 epigenetic drugs. The screening approach is outlined in Figure 1A. We used PSN1, DanG, and PaTu8988T cells as models with high MYC expression and Panc1, HPAC, and PaTu8988S cells as models with low MYC expression. MYC-high models showed significantly increased MYC protein expression (Figure 1B and Supplemental Figure 1A; supplemental material available online with this article; https://doi.org/10.1172/jci.insight.151353DS1) and enrichment of MYC signatures in gene set enrichment analysis (GSEA; Figure 1C). For the screening experiment, a 7-point drug dilution was used, and the experiment was conducted as 3 technical replicates. Using this experimental approach (Supplemental Table 1), we observed increased activity of several histone deacetylase inhibitors in PDACs with increased MYC expression (Figure 1D and Supplemental Table 2), which is consistent with our recent screening efforts using an FDA-approved drug library (20) and data of the DoRothEA database (23). This database connects drug activity of 265 compounds determined in 1000 cell lines to the activity of transcription factors (23). In addition, the activity of the chemotherapeutics mitomycin C and gemcitabine was found to be connected to MYC (Figure 1D). Again, gemcitabine was detected to trigger an MYC-associated vulnerability in our recent screen (20), and the efficacy of both chemotherapeutics was associated with MYC in the DoRothEA database (23). In addition, the JAK2 inhibitor XL019, the PIM kinase inhibitor AZD1208, and PRMT5i GSK591 were defined as potentially novel hits in the screening experiment (Figure 1D and Supplemental Table 2).

### PRMT5 is connected to MYC in PDAC.

Given our overall aim to find and characterize an epigenetic MYC-associated vulnerability in the context of PDAC, we investigated the GSK591 target PRMT5. PRMT5 is a type II protein arginine methyltransferase catalyzing symmetrical dimethylation of arginines in histones and other proteins (24). High PRMT5 expression was recently linked to worse survival of patients with PDAC (25–27). To underpin the connection of MYC to PRMT5, we analyzed curated mRNA expression data sets (20, 28). PDACs with high expression of the *PRMT5* mRNA were analyzed with GSEA using the GeneTrail 3.0 web tool. In 2 data sets, PDACs with high expression of PRMT5 were characterized by an active MYC network (Figure 1E and Supplemental Table 3). The activation of the MYC network in PDAC cells with high PRMT5 expression was also observed in conventional human cell lines (Supplemental Figure 1B), primary human patient-derived cell lines (PDCLs; Supplemental Figure 1C and Supplemental Table 3), patient-derived organoids (PDOs; Supplemental Table 3), and primary murine PDAC cell lines (Supplemental Figure 1D), and the interaction of the proteins was supported by a STRING analysis (Supplemental Figure 1E). Gene signatures modulated in PDAC models with high PRMT5 expression are illustrated in Supplemental Table 3 and include significant RNA-splicing, DNA repair, ribosome, metabolic, and cell cycle/mitosis signatures. In conventional human PDAC cell lines, a positive correlation of *MYC* and *PRMT5* mRNA expression was observed (Figure 1F). To place this finding into context, we analyzed the correlation of *MYC* and *PRMT5* mRNA expression across tumor entities. Although the correlation was detected in solid and hematopoietic cancers, the highest correlation coefficient for both factors was detected in PDAC (Figure 1G). In addition, *MYC* mRNA correlated with the mRNA expression of other enzymes of the *PRMT* family (Supplemental Figure 1F). To corroborate the correlation of PRMT5 and MYC at the protein level, we determined MYC and PRMT5 expression by Western blotting in PDAC cell lines and observed a positive correlation (Figure 1B and Supplemental Figure 1G). Accessing MYC and PRMT5 protein expression of human PDAC cell lines via the DepMap portal (https://depmap.org/portal/) showed a lower positive correlation coefficient, which was not statistically significant, however (Supplemental Figure 1H). To find evidence that PRMT5 is a relevant therapeutic target, we accessed data of a CRISPR/Cas9 dropout screen for the PDAC context (*n* = 23) (https://score.depmap.sanger.ac.uk/) (2). We found 87% of the PDAC lines had significantly impaired fitness upon genetic inactivation of *PRMT5* (Supplemental Figure 1I). The lines with high MYC expression used in the pharmacological screening experiment are marked in Supplemental Figure 1I and demonstrated loss-of-fitness scores below the mean. The lines used in the epigenetic drug screening harboring low MYC expression were not included in this particular CRISPR/Cas9 dropout screen (2). In addition, we accessed the CRISPR/Cas9 dropout data of the DepMap portal (https://depmap.org/portal/) (29). Again, most PDAC cell lines (*n* = 46) showed a strong PRMT5 gene effect (Supplemental Figure 1J). Gene effect scores for PRMT5 and MYC were not correlated (Supplemental Figure 1J). In sum, PRMT5 is a therapeutic target in PDAC, and high mRNA expression of the arginine methyltransferase marks cancers with high MYC expression and network activity.

### MYC induces PRMT5 expression.

**Figure 2:**
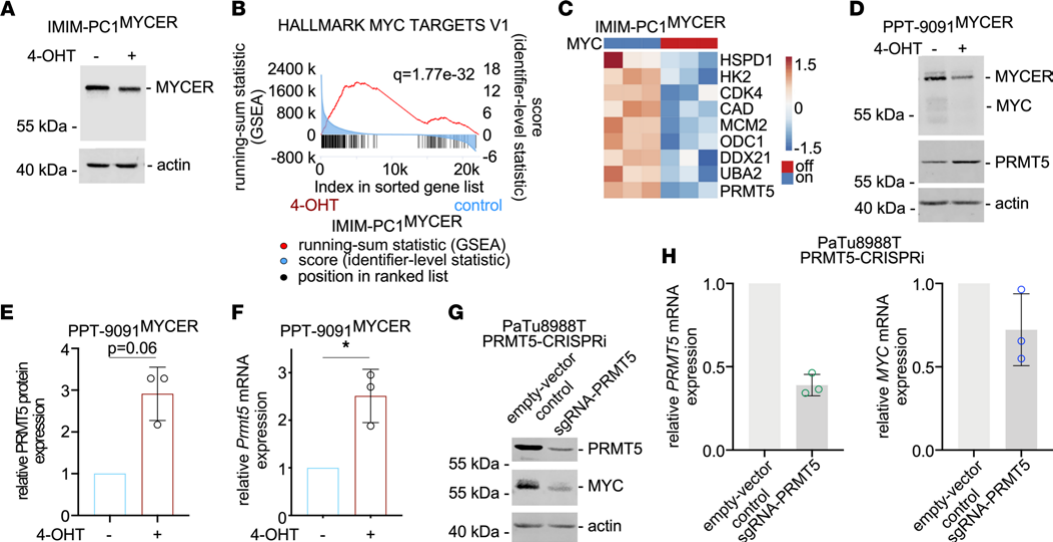
MYC controls PRMT5 expression. (**A**) Western blot showing protein expression of MYC^ER^ and β-actin (loading control) in human IMIM-PC1^MYC-ER^ cells treated with 4-hydroxytamoxifen (4-OHT) (48 hours, 600 nM) or vehicle control (EtOH). One representative experiment out of 3 is shown. (**B**) GSEA of RNA-Seq expression data from human IMIM-PC1^MYC-ER^ cells treated with 4-OHT to activate MYC (24 hours, 500 nM) or vehicle control. Depicted is the HALLMARK signature MYC TARGETS V1, including the *q* value. (**C**) Heatmap of selected MYC target genes in human IMIM-PC1^MYC-ER^ cells treated as in **B**. Adjusted *P* value of all shown genes; *P* < 0.05. Data are based on the RNA-Seq described in **B**. (**D**) Western blot showing protein expression of MYC, MYC^ER^, PRMT5, and β-actin (loading control) in murine PPT-9091^MYC-ER^ PDAC cells treated with 4-OHT (48 hours, 600 nM) to activate MYC or left as vehicle control. One representative experiment out of 3 is shown. (**E**) Quantification of 3 independent experiments from **D**; *P* value of a paired 2-tailed *t* test is depicted. (**F**) Quantification of *Prmt5* mRNA expression of murine PPT-9091^MYC-ER^ PDAC cells treated with 4-OHT (48 hours, 600 nM) or vehicle control determined out of 3 biological replicates performed as technical triplicates by qPCR. *GAPDH* was used to normalize the expression. *P < 0.05; paired 2-tailed *t* test. (**G**) PaTu8988T control or PRMT5 CRISPRi cells were analyzed. Western blot of PRMT5 and MYC expression. β-Actin: loading control (*n* = 2). (**H**) PaTu8988T control or PRMT5 CRISPRi cells were analyzed for mRNA expression of *PRMT5* (left panel) or *MYC* (right panel). Three biological replicates performed as technical triplicates were analyzed. *GAPDH* was used to normalize the expression. *P < 0.05; paired 2-tailed *t* test.

To test a direct connection of MYC to PRMT5, we used conditional models based on an MYC estrogen receptor fusion protein (MYC^ER^). The human PDAC cell line IMIM-PC1 is characterized by low expression of endogenous MYC (18). Expression of MYC^ER^ in IMIM-PC1 cells results in downregulation of endogenous MYC expression, as described recently (18). Therefore, only the fusion protein is detected in Western blots (Figure 2A). Analyzing RNA-Seq data of IMIM-PC1^MYCER^ cells treated with 4-hydroxytamoxifen (4-OHT) with GSEA demonstrated activation of an MYC-directed program (Figure 2B). In addition, *PRMT5* mRNA expression was significantly induced in 4-OHT–treated IMIM-PC1^MYCER^ cells (Figure 2C). To investigate the direct connection of MYC to PRMT5 across species, we used a murine PDAC cell line with low endogenous MYC expression and transduced the cells with an MYC^ER^ expression vector, PPT-9091^MYCER^ (Figure 2D and ref. 20). Also, in PPT-9091^MYCER^ cells, 4-OHT treatment induced PRMT5 expression at the protein (Figure 2, D and E) and mRNA level (Figure 2F). To test the connection of MYC and PRMT5 reciprocally, we used enhanced CRISPR interference (CRISPRi) mediated by dual repressor domains based on Krüppel-associated box (KRAB) and the repressive domain of methyl CpG-binding protein 2 (MeCP2), fused to an inactive Cas9 (30). A guide RNA targeting the *PRMT5* gene reduced PRMT5 protein and mRNA expression (Figure 2, G and H). Interestingly, MYC expression was reduced at both levels (Figure 2, G and H), although the reduction at the mRNA level was variable and modest. Together, these data showed that MYC can induce the expression of PRMT5 in PDAC.

### MYC controls PRMT5i activity.

**Figure 3:**
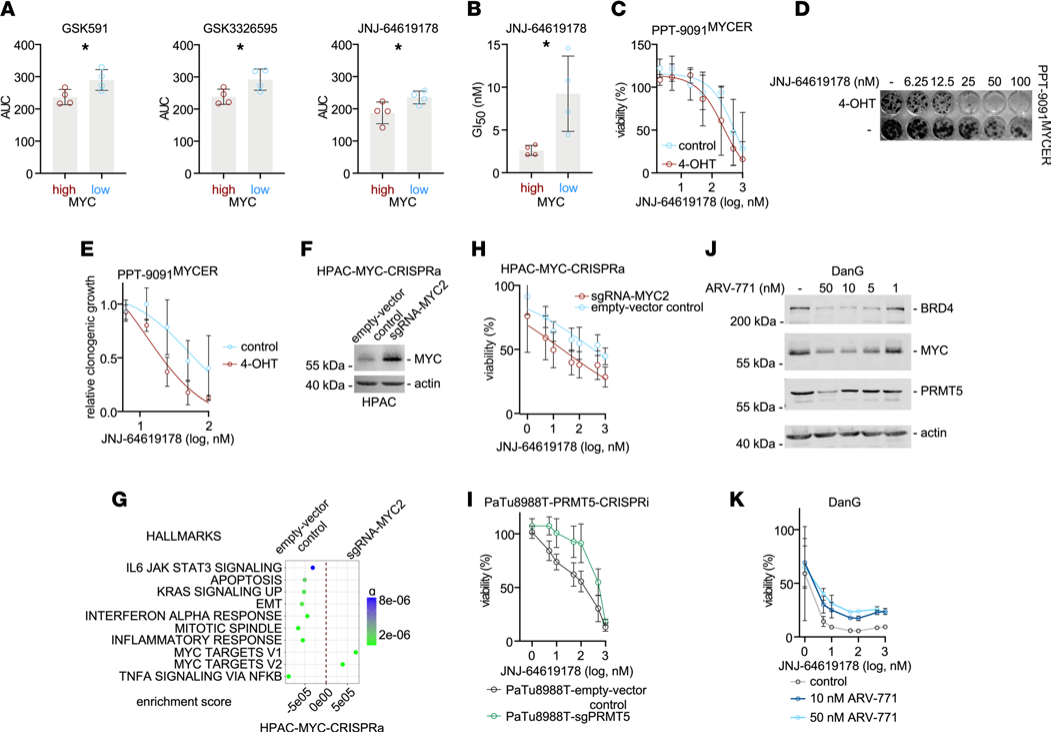
PRMT5i response is tuned by Myc. (**A**) Viability of MYC-high (DanG, PSN1, PaTu8988T, HUPT3) and MYC-low (Panc1, PaTu8988S, HPAC, Panc0504) cell lines treated for 72 hours with the indicated compounds, measured by CellTiter-Glo assay; *n* = 3, dosage range of 2 nM–10 μM used to determine AUC. *P < 0.05; unpaired 2-tailed *t* test. (**B**) Growth inhibitory 50% (GI_50_) concentrations of cell lines described in **A** after 7 days of treatment with JNJ-64619178. Clonogenic growth–based dose-response curves were analyzed with a nonlinear regression for curve fitting. *n* = 3, *P < 0.05; unpaired 2-tailed *t* test. (**C**) Dose-response curves of PPT-9091^MYC-ER^ cells with 4-OHT (600 nM) or vehicle control after 6 days of treatment with JNJ-64619178. Viability was measured by CellTiter-Glo assay. (**D**) Clonogenic growth assay of PPT-9091^MYC-ER^ cells with 4-OHT (600 nM) or vehicle (EtOH) after 7 days of treatment with JNJ-64619178. One representative experiment is depicted. (**E**) Quantification of 3 independent biological replicates of **D**. (**F**) Control and MYC-CRISPRa HPAC cells were analyzed by Western blot for MYC expression; β-actin: loading control (*n* = 4). (**G**) RNA-Seq of control and MYC-CRISPRa HPAC cells analyzed by GSEA using the GeneTrail platform. Enrichment scores and *q* value shown. (**H**) JNJ-64619178 dose-response curves of control and MYC-CRISPRa HPAC cells. Cells were treated for 6 days and ATP was measured as surrogate, *n* = 3. (**I**) JNJ-64619178 dose-response curves of control and PRMT5-CRISPRi PaTu8988T cells. Cells were treated for 6 days, and ATP was measured as surrogate. *n* = 4. (**J**) DanG cells were treated with ARV-771 (72 hours) as indicated or left as vehicle-treated controls. Western blotting demonstrated expression of BRD4, MYC, and PRMT5. β-Actin: loading control. One representative experiment out of 3 is shown. (**K**) JNJ-64619178 dose-response curve of DanG cell, cotreated with vehicle control or ARV-771 as indicated, *n* = 3.

For the validation of the screening results, we used 4 lines with high MYC expression (HUPT3, PaTu8988T, PSN1, and DanG) and 4 lines with low MYC expression (Panc1, PaTu8988S, HPAC, and Panc0504). We investigated the response to 3 PRMT5i: GSK591 (31), GSK3326595 (32), and JNJ-64619178 (33). We recapitulated the screen by the use of a 7-point drug dilution with the inhibitors and measurement of cellular ATP as a surrogate for the dose response. We treated the cells for 72 hours and determined the area under the dose-response curves (AUC). For all PRMT5i, the mean AUC was significantly lower in PDACs with high MYC expression (Figure 3A), validating the screen. Interestingly, we detected that the PRMT5i induced a slow cellular response with augmented cell killing upon longer drug exposure times (Supplemental Figure 2A), which might contribute to the rather small difference in the AUC observed after 72 hours (Figure 3A). Since the JNJ-64619178 half-maximal growth-inhibitory concentration (GI_50_) in sensitive cells was in the single-digit nanomolar range (Supplemental Figure 2B), we repeated the validation experiments by a) using a long-term clonogenic growth assay and b) using JNJ-64619178, the inhibitor with the lowest GI_50_. As shown in Supplemental Figure 2C, cells with high MYC expression revealed augmented inhibition of clonogenic growth upon JNJ-64619178 treatment. We quantified the clonogenic assays and used the dose-response curves (Supplemental Figure 2D) to determine the GI_50_ values by a nonlinear regression. Although the GI_50_ values in PDAC cell lines with low MYC expression revealed a high range, the mean GI_50_ values for cells with high MYC expression were significantly lower (Figure 3B). Furthermore, the JNJ-64619178 GI_50_ values correlated with the *PRMT5* gene effects of the CRISPR dropout screen. However, the correlation was not statistically significant (Supplemental Figure 2E).

To further substantiate the direct control of the PRMT5i sensitivity by MYC and to cope with the intertumoral heterogeneity of drug responses, we used the conditional MYC overexpression model. Activation of MYC by treating PPT-9091^MYC-ER^ cells with 4-OHT reduced the JNJ-64619178 GI_50_ value from 713 to 241 nM (Figure 3C). Consistently, clonogenic growth inhibition by JNJ-64619178 was augmented by MYC activation (Figure 3, D and E). To investigate the connection of increased MYC expression to PRMT5i sensitivity across species, we used human HPAC cells, which are characterized by low MYC expression. Since MYC protein expression involves control mechanisms integrated by the 3′-UTR of the *MYC* mRNA and such control is engaged by cellular stress pathways (34), we aimed to establish a model where MYC expression is driven from the endogenous promoter. Therefore, we used CRISPR activation (CRISPRa) by dCas9-synergistic activation mediator (dCas9-SAM) system (35). An sgRNA targeting the *MYC* gene induced MYC protein expression in HPAC cells 3-fold (Figure 3F and Supplemental Figure 3A) and *MYC* mRNA expression 2.6-fold (Supplemental Figure 3B). In GSEA of RNA-Seq data of these cells, we also detected the MYC network to be activated (Figure 3G). Also, *PRMT5* mRNA expression was slightly higher in HPAC cells with increased MYC expression (Supplemental Figure 3C). Functionally, MYC overexpression resulted in increased proliferation (Supplemental Figure 3D). As in the conditional model, HPAC cells were sensitized to JNJ-64619178 by MYC expression (Figure 3H), consistent with a reduction in the calculated GI_50_ values from 347 nM to 22 nM for JNJ-64619178‚ respectively. To address the regulation of the PRMT5i sensitivity in a PRMT5 loss-of-function model, we used the PRMT5 CRISPRi PaTu8988T cells, which downregulated PRMT5 and MYC (Figure 2, G and H). Here, reduced sensitivity to JNJ-64619178 in PRMT5 CRISPRi cells was observed (GI_50_ values 104 nM versus 513 nM, respectively) (Figure 3I). These data substantiate the role of MYC in tuning the PRMT5i response. The reduced PRMT5 expression in the model was not connected to an altered proliferative capacity (Supplemental Figure 3E), supporting the notion that not proliferation but a specific vulnerability controlled by an MYC-PRMT5 module is responsible for the altered sensitivity. In addition, we used a pharmacological loss-of-function model by indirect targeting of MYC with a bromodomain and extraterminal motif (BET) protein degrader. The BET degrader ARV-771 (36) efficiently degraded BRD4 in DanG cells (Figure 3J). Consistent with the well-described function of BRD4 to maintain MYC expression, the oncogene was downregulated in a dose-dependent fashion (Figure 3J). At the highest concentration of ARV-771, PRMT5 expression was also decreased. Importantly, ARV-771–mediated decreased MYC expression was connected to reduced efficacy of JNJ-64619178 (Figure 3K). Analyzing the drug interaction using the SynergyFinder 2.0 platform (37) and a zero interaction potency (ZIP) model (38) resulted in a negative ZIP score, demonstrating antagonistic action of JNJ-64619178 and ARV-771 (Supplemental Figure 3F). Therefore, in different species and models, MYC modulated PRMT5i sensitivity.

### A PRMT5i-sensitive subtype in PDO and PDCL.

**Figure 4:**
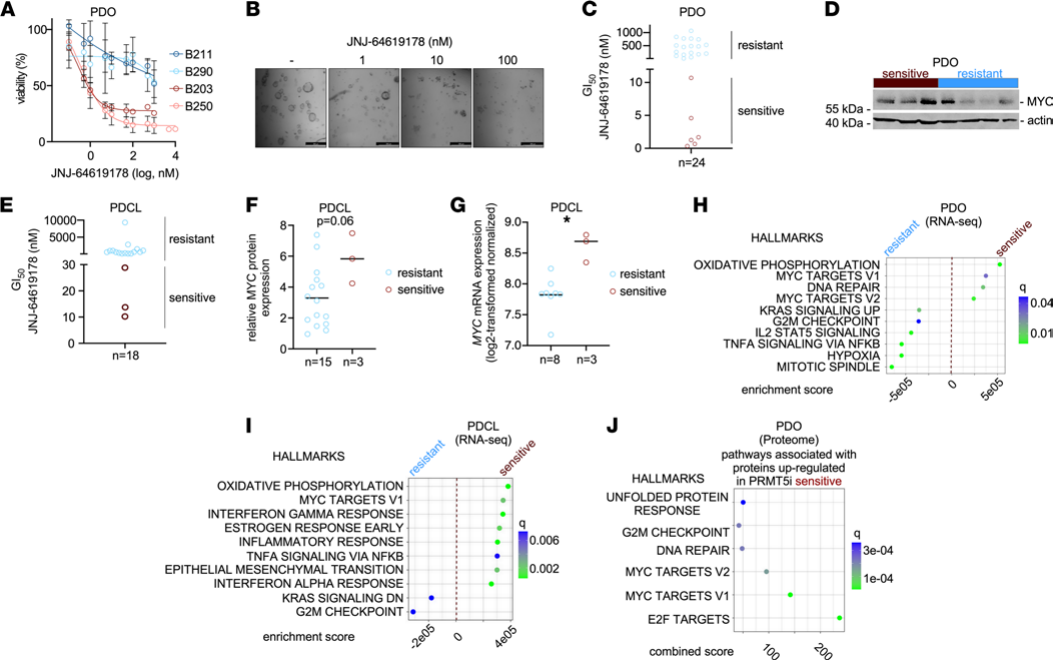
PRMT5i response in primary human PDAC models. (**A**) Dose-response curves of 4 human PDOs after 6 days of treatment with JNJ-64619178. Viability was determined with CellTiter-Glo assay. Sensitive: red, resistant: blue. (**B**) Microscopy of a sensitive organoid treated with the indicated dose of JNJ-64619178 over 6 days. Scale bar: 500 μM. (**C**) GI_50_ values of 24 human PDOs were calculated. Sensitive: red, resistant: blue. (**D**) MYC expression was analyzed in selected PDOs (*n* = 7) by Western blot. β-Actin: loading control. Sensitive: red, resistant: blue. One Western blot was performed. (**E**) GI_50_ values of 18 primary PDCLs were calculated. Sensitive: red, resistant: blue. (**F**) Relative MYC protein expression, determined by Western blot, in sensitive (*n* = 3, red) and resistant (*n* = 15, blue) PDCL was compared. *P* value of Mann-Whitney *U* test is depicted. MYC Western blot was performed once. (**G**) MYC mRNA expression based on RNA-Seq in JNJ-64619178–sensitive and –resistant PDCLs. *P < 0.05; Mann-Whitney *U* test. (**H**) JNJ-64619178 GI_50_ values of PDOs were grouped into quartiles and differentially expressed genes of most sensitive (first quartile, *n* = 6) and most resistant (fourth quartile, *n* = 6) PDOs were calculated. The log-fold change was used as a rank to perform a preranked GSEA. Depicted are the top 10 HALLMARK signatures; *q* value is color coded. (**I**) JNJ-64619178 GI_50_ values of PDCL were grouped into quartiles and analyzed corresponding to **H**. Sensitive: first quartile, *n* = 3, resistant: fourth quartile, *n* = 3. Depicted are the top 10 HALLMARK signatures; *q* value is color coded. (**J**) Proteomics-based protein expression of PRMT5i-sensitive (first quartile, *n* = 6) and -resistant (fourth quartile, *n* = 6) PDOs was used to determine differentially expressed proteins. All proteins upregulated in sensitive PDOs were analyzed using the Enrichr web tool. Combined scores of the HALLMARK signatures with an adjusted *P* value and *q* < 0.05 are shown.

To investigate the MYC-directed control of PRMT5i responsiveness in a human model with potential predictability for the clinical behavior of PDACs (39–43), we used organoid pharmacotyping. Consistent with the 2D cellular models, prolonged incubation periods with JNJ-64619178 increased the response of the organoids. Therefore, we treated a panel of PDOs with JNJ-64619178 over 6 days. The dose-response curves of 2 very sensitive and 2 very resistant organoids are shown in Figure 4A. Treatment changed the normal organoid growth, and small, irregularly shaped organoids were observed (Figure 4B). Even after a prolonged treatment period of 2 weeks, the difference between a sensitive and a resistant PDO remained (Supplemental Figure 4A). In 24 investigated PDOs, again a PRMT5i-sensitive subtype was detected, including PDOs with GI_50_ values in the single-digit nanomolar range (Figure 4C). In Western blot analysis of selected PDOs, the highest MYC protein expression was observed in an organoid belonging to the PRMT5i-sensitive PDAC subtype (Figure 4D). However, we also observed resistant organoids with high MYC protein expression (Figure 4D).

Recently, the methylthioadenosine phosphorylase gene (*MTAP*), which is frequently codeleted with *CDKN2A*, was demonstrated to control sensitivity to PRMT5i EZP015556 in the PDAC context (40). However, JNJ-64619178–sensitive PDO lines exist, which express *MTAP* mRNA (Supplemental Figure 4B), consistent with a recent observation (33). To further corroborate the findings from the 3D models, we used 18 PDCLs, which were documented to be genetically stable and a valid model to study drug responses (44). Also in this model, we were able to demonstrate the existence of a PDAC subtype that was more sensitive to PRMT5i (Figure 4E). We determined MYC protein expression by Western blot in the PDCLs and observed a negative correlation between MYC protein expression and JNJ-64619178 GI_50_ values (Spearman’s *r* = –0.52, *P* = 0.03; Pearson’s *r* = –0.32, *P* > 0.5). The mean MYC protein expression was higher in PRMT5i-sensitive PDCLs, although resistant lines with high MYC expression were detected (Figure 4F). We accessed RNA-Seq data, available for 11 of the investigated PDCL lines. As in the PDOs, *MTAP* mRNA expressing PRMT5i-sensitive PDCL lines exist (Supplemental Figure 4C). Fitting to the increased MYC protein expression, PRMT5i-sensitive PDCL lines expressed higher *MYC* mRNA (Figure 4G). In PDCLs, PRMT5i-sensitive lines tended to show lower *PRMT1* mRNA expression (Supplemental Figure 4C). Considering that PRMT1 might compensate PRMT5 functions, we tested the combination of a PRMT5i and a PRMT1i. Indeed, we observed synergism of both inhibitors (Supplemental Figure 4, D and E), which is consistent with a report from 2019 (45).

Next, we analyzed RNA-Seq data of the PDOs and PDCLs. In both cellular models, we grouped the GI_50_ values in quartiles and calculated differentially expressed genes of the lines belonging to the most sensitive and most resistant quartile (Supplemental Table 4). We used the log-fold change as a rank and performed a preranked GSEA. In PDOs (Figure 4H) and PDCLs (Figure 4I), we detected enrichment of MYC signatures in sensitive lines. In addition, we calculated differentially expressed genes between lines belonging to the most sensitive quartile and all others. Again, the log-fold change was used for a preranked GSEA. Here, MYC signatures were not enriched in the sensitive lines (Supplemental Figure 4, F and G), which is explained by the existence of PDACs with high MYC activity but low PRMT5 sensitivity. This finding is consistent with the MYC expression analysis in these phenotypes (Figure 4, D and F).

Furthermore, we used proteomes of PRMT5i-sensitive organoids. We again compared the PDOs of the most sensitive to the most resistant quartile and calculated differentially expressed proteins (Supplemental Table 4). We analyzed the proteins upregulated in the sensitive PDOs using the Enrichr web tool (46). Here, both MYC HALLMARK signatures were associated with proteins upregulated in sensitive PDOs (Figure 4J). In addition, Kyoto Encyclopedia of Genes and Genomes (KEGG) pathway analysis of upregulated proteins demonstrated enrichment of splicing and DNA repair signatures in sensitive organoids (Supplemental Figure 4H). Combined, these results indicate a PRMT5i-sensitive subtype exists in primary 2D and 3D models of PDAC.

### PRMT5i induces apoptosis in PDAC cells with high MYC expression.

**Figure 5:**
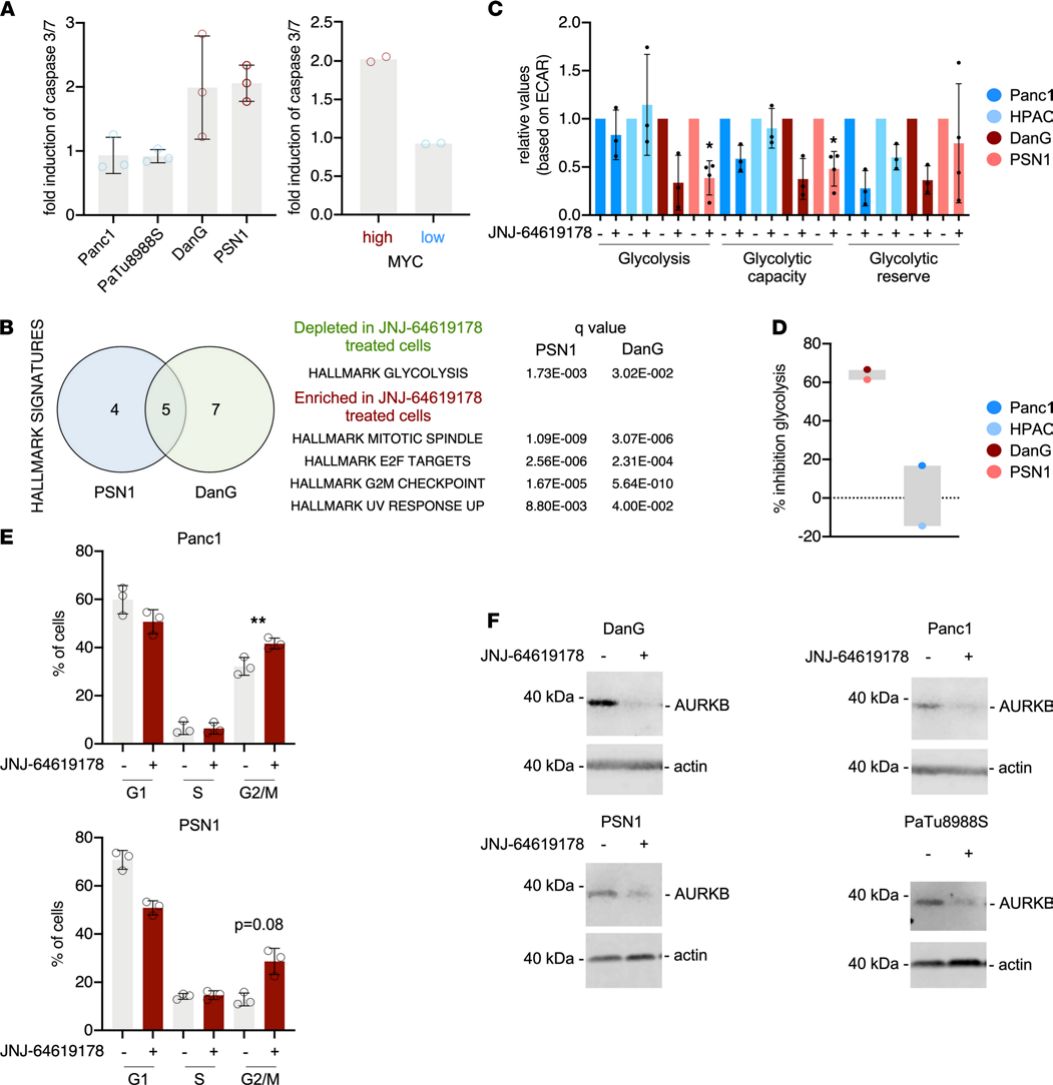
PRMT5 inhibition induces apoptosis and alters metabolism in PDAC cells with deregulated MYC. (**A**) Activity of caspase-3/7 in 2 MYC-high (DanG, PSN1) and 2 MYC-low (Panc1, PaTu8988S) cell lines after treatment with 40 nM JNJ-64619178 for 3 days. Left panel: fold induction of caspase-3/7 in each line, 3 biological replicates per line were performed; right panel: mean fold induction of caspase-3/7 of the 2 MYC-high and 2 MYC-low lines. (**B**) RNA-Seq of untreated and JNJ-64619178–treated (3 days, 20 nM) DanG and PSN1 cells analyzed with GSEA and the HALLMARK signatures. Signatures with a *q* less than 0.05 were investigated in a Venn diagram. The 5 signatures modulated by JNJ-64619178 in both lines, including their *q* value, are depicted. (**C**) Cell lines with low MYC expression (Panc1 and HPAC, blue) and high MYC expression (DanG and PSN1, red) were treated with 20 nM JNJ-64619178 for 3 days. Extracellular acidification rate (ECAR) values were measured via the Seahorse assay and used to calculate glycolysis, glycolytic capacity, and glycolytic reserve. *P < 0.05; unpaired 2-tailed *t* test (*n* ≥ 3). (**D**) Percentage inhibition of glycolysis based on the ECAR values from **C**. (**E**) Cell cycle distribution of the indicated cell lines (MYC high: PSN1; MYC low: Panc1). Cell cycle distribution was determined by FACS of propidium iodide–stained cells. Cells were treated with 20 nM JNJ-64619178 or DMSO for 4 days. Results of 3 biological replicates are shown. **P < 0.01; *P* value in PSN1 cells is depicted; paired 2-tailed *t* test. (**F**) Aurora kinase B (AURKB) Western blot analysis of indicated cell lines treated for 4 days with 20 nM JNJ-64619178 or left as a vehicle-treated control. β-Actin: loading control. One representative experiment out of 2 replicates is depicted.

After validating that PRMT5i triggers an MYC-associated vulnerability, we investigated the underpinning mechanism. JNJ-64619178 distinctly reduced the symmetrical dimethylation of histone H4R3 (Supplemental Figure 5A). Recent data demonstrated that PRMT5 blockade induces a DNA damage response in PDAC cells (27). Therefore, we investigated phosphorylation of H2AX as a surrogate for the DNA damage response. Although phosphorylation of H2AX occurred in response to PRMT5 inhibition, it was induced independently of the MYC expression status (Supplemental Figure 5A). In contrast, induction of apoptosis, which was investigated by monitoring the cleavage of the caspase substrate PARP (Supplemental Figure 5A) and determining activity of executioner caspase-3 and caspase-7 (Figure 5A), was connected to cells with high MYC expression. In addition, downregulation of MYC by the BET degrader ARV-771 significantly reduced JNJ-64619178–mediated induction of caspase activity, as evidenced by cleavage of PARP (Supplemental Figure 5B). To find relevant pathways, we analyzed RNA-Seq of JNJ-64619178–treated DanG and PSN1 cells with GSEA. We observed an overlap of 5 HALLMARK signatures consistently modulated by JNJ-64619178 (Figure 5B). HALLMARK signatures connected to the G2/M phase of the cell cycle and mitosis as well as to the pro-proliferative E2F pathway were enriched in JNJ-64619178–treated DanG and PSN1 cells (Figure 5B). In contrast, a signature connected to glycolysis was inhibited by JNJ-64619178 (Figure 5B). JNJ-64619178–modulated REACTOME and KEGG signatures can be found in Supplemental Table 5. Although epithelial-mesenchymal transition signatures were depleted in PRMT5i-treated PSN1 cells (Supplemental Table 5) and PRMT5 was linked to epithelial-mesenchymal transition (26), vimentin and E-cadherin expression were not changed in JNJ-64619178–treated PSN1 and DanG cells (Supplemental Figure 5C). We next confirmed effects of the PRMT5i on glycolysis and the cell cycle. Glucose uptake was especially reduced in the PDAC lines with high MYC expression (Supplemental Figure 5D). This was corroborated by Seahorse analysis, which demonstrated an impact of JNJ-64619178 on glycolysis, especially in lines with high MYC expression (Figure 5, C and D). Irrespective of the MYC status, treatment with JNJ-64619178 arrested the cell cycle in the G2/M phase (Figure 5E), which was also observed by the investigation of the expression of mitotic marker genes, like targeting protein for Xklp2 (TPX2) (Supplemental Figure 5E), which functions in assembly of the mitotic spindle. Since Aurora kinase B (AURKB) was demonstrated to be regulated by PRMT5 (47), we investigated expression by Western blot. In contrast to TPX2, we detected decreased expression of AURKB upon PRMT5 inhibition in all investigated PDAC lines (Figure 5F). In sum, these data showed that the cellular response toward PRMT5i was switched to apoptosis in selected PDAC cells with high MYC expression.

## Discussion

In this study, we unbiasedly found PRMT5i to trigger an MYC-associated epigenetic vulnerability. We describe a robust connection of MYC to PRMT5 in human and murine PDAC cells and observed across models a highly PRMT5i-sensitive PDAC subtype.

By controlling transcriptional regulation, the epigenetic landscape, mRNA processing and splicing, or oncogenic signaling pathways, PRMT5 plays a crucial role in tumor maintenance (24, 48). The enzyme belongs to a group of arginine methyl transferases, which have been subdivided into type I (PRMT1, 2, 3, 6, 8 and CARM1), type II (PRMT5, PRMT9), and type III (PRMT7) enzymes (24, 48). Type I PRMTs monomethylate and asymmetrically dimethylate arginine, type II enzymes monomethylate and symmetrically dimethylate arginine, and the type III enzyme monomethylates arginine. PRMT1 was recently shown to be a potential therapeutic target in PDAC (45, 49). However, in cases in which PRMT1 inhibitors were combined with chemotherapy, like gemcitabine, clear evidence demonstrates the need to stratify for responders (50). PRMT5 is the primary type II molecule methylating arginines of histones and other proteins. In the context of PDACs, PRMT5 was linked to EMT (26) and glycolysis (25), 2 processes associated with the more aggressive basal like subtype of PDAC (20, 51). Furthermore, genetic or pharmacological inhibition of PRMT5 sensitizes PDAC cells toward gemcitabine (27), offering an opportunity for translation. Consistently, PDACs with high PRMT5 expression demonstrate a worse prognosis (25–27). The robust connection of PRMT5 to MYC, a transcription factor whose activity is also enriched in basal-like PDACs (20, 51), supports the notion that PRMT5 is a relevant target in an aggressive PDAC subtype, with high therapeutic resistance to currently used chemotherapies (52).

PRMT5i, like JNJ-64619178 (ClinicalTrials.gov NCT03573310), PF-06939999 (ClinicalTrials.gov NCT03854227), and GSK3326595 (ClinicalTrials.gov NCT02783300), entered clinical testing in advanced solid cancers and hematological malignancies. Consistent with our analysis of patient-derived PDAC models, recent pharmacotyping of human PDAC organoids pointed to a relevant PRMT5i-sensitive subtype (40). In part, the high potency of the PRMT5i EZP015556 in PDAC is due to the frequent *CDKN2A*-associated codeletion of *MTAP*, an enzyme of the methionine salvage pathway (48). *MTAP* deficiency induces the accumulation of 2-methylthioadenosine (MTA), which is an endogenous inhibitor of PRMT5. This molecular circuit also contributes to higher activity of PRMT1 inhibitors in *MTAP*-deficient cancer cells (45). Restoring MTAP expression in deficient PDAC organoids distinctly decreased EZP015556 sensitivity, and MTA metabolite abundance negatively correlated with GI_50_ values (40). However, a relevant subgroup of EZP015556-sensitive PDAC organoids has been described, which are *MTAP* proficient (40). We observed JNJ-64619178–sensitive PDOs and PDCLs that expressed *MTAP* mRNA. These data are in line with observations made by Brehmer et al., whose explanation for MTAP-expressing, sensitive cancer cells is the mode of inhibition of PRMT5 by JNJ-64619178, which binds to the S-adenosylmethionine- and the substrate-pocket (33).

Considering further markers for PRMT5i sensitivity, mutations in splicing factors (53) or gene signatures indicative for the addiction to the splicing machinery (32, 47, 54) might be relevant. However, PRMT5i sensitivity connected to the splicing machinery is context dependent and relevant in acute myeloid leukemia (33). In addition, work from 2018 conducted in a large cell line panel showed that the mutational status of the tumor suppressor p53, whereby mutations align with resistance, is a predictor for PRMT5i responsiveness (32). Markers defined by others, including MYC expression (55), the CLNS1A/RIOK1 expression ratio (47), or MTAP deletion status (56–58), were not connected to PRMT5 sensitivity in this large cell line panel study (32). Different tumor entities included in the investigated lines might be a confounder contributing to oversee tumor entity–specific markers. Underscoring this concept, we observed a different degree of the MYC-PRMT5 connection across tumor entities, supporting the notion that entity-specific predictors should be developed.

Our work suggests that deregulated MYC generates a PRMT5 dependency. This notion is especially supported by our genetic gain- and loss-of-function experiments. However, the heterogeneity observed in the primary PDAC models, with the existence of PDACs with high MYC expression and JNJ-64619178 resistance, points to the complexity of the MYC network, whose oncogenic potency is known to be regulated by several cofactors (59). Additionally, such data demonstrate the need for multivariate predictors to more precisely determine the PRMT5i-sensitive state in PDACs with high MYC expression.

PaTu8988T, a line included in our analysis as a model with high MYC expression, was determined to be the most GSK3203591-resistant cell line in a panel of 20 PDAC lines (32). This contrasts with the data of our clonogenic growth assays. PaTu8988T cells showed a significant loss of fitness upon the genetic inhibition of PRMT5 (2), but the CRISPRi-dependent reduction in PRMT5 expression can be compensated. Whether different assay types or the extent or modes of inhibition contribute to the discrepant results remains to be determined.

We defined a robust connection of PRMT5 to MYC in PDAC. Consistently, PRMT5 is a direct MYC target gene in the *Eμ-myc* lymphoma (55) and liver cancer models (60). We detected induction of PRMT5 in MYC conditional human and murine PDAC models after activation of the oncogene. Furthermore, PDACs with high PRMT5 expression enrich MYC signatures. Genetic inactivation of PRMT5 preferentially induced death of B cells from *Eμ-myc* mice compared with wild-type B cells (55), demonstrating the dependency of cells with deregulated MYC on PRMT5. A conserved function of PRMT5, which is relevant for its oncogenic activity, is the methylation of proteins involved in mRNA splicing (24, 47, 48). It was proposed that cancer cells with high MYC activity, which leads to an increased total RNA burden, rely on PRMT5 to orchestrate splicing fidelity (55). Supportively, an unbiased genetic screen in human mammary epithelial cells found the *BUD31* gene, a spliceosome component, to be synthetically lethal with MYC (15). Subsequent work demonstrated that the blockade of the spliceosome leads to intron retention and death of MYC-hyperactivated breast cancers (61). Additionally, T-025, an inhibitor of CDC2-like kinases, which control mRNA splicing, induces alternative splicing and death of MYC-hyperactivated cancer cells (62). The established function of PRMT5 to ensure splicing fidelity and dependency of MYC-deregulated cells on the splicing machinery contribute to the increased sensitivity of PDAC cells with deregulated MYC expression to PRMT5i.

Interestingly, reduced splicing fidelity exposes an additional MYC-associated vulnerability. PRMT5 blockade was shown to prevent the removal of detained introns in pro-proliferative genes, including the mitotic regulator AURKB (47). Cancer cells with deregulated MYC are specifically characterized by a mitotic vulnerability (19), and AURKB was demonstrated to be synthetically lethal with MYC (63). Further, in glioblastoma, PRMT5i-induced reduction in the splicing fidelity affects pathways important for cell cycle progression (54), underscoring our findings. Therefore, it is rational to also consider the contribution of cell cycle and mitotic genes, like AURKB, to explain increased PRMT5i sensitivity of PDAC cells with high MYC expression.

PRMT3 is included in the MYC HALLMARK V2 gene signature. Interestingly, we observed a positive correlation of the expression of several PRMTs with MYC. Such data might indicate the strong dependency of MYC-driven cancers on the arginine methylation machinery. Whether such a multilayer buffering system contributes to the heterogeneity of the PRMT5i sensitivity of cancers with high MYC expression remains to be deciphered in future work. Multilayer buffering systems may also require targeting more than 1 PRMT to tackle MYC-driven cancers, a further consideration that needs to be extended in the future in more detail.

In vivo efficacy of JNJ-64619178 was recently demonstrated in patient-derived xenotransplant models of hematological malignancies and solid cancers, including PDAC (33). Together with our data, including the connection of PRMT5i sensitivity to MYC-hyperactivated PDACs, this supports the conclusion to further develop PRMT5i-based therapies for PDAC. However, additional preclinical research is needed, including a more precise molecular understanding of PRMT5i sensitivity and the development of combinatory therapies to advance PRMT5i to clinical testing in PDAC.

## Methods

### Chemicals.

The 4-OHT was purchased from MilliporeSigma; the DMSO for cell culture was from AppliChem; JNJ-64619178, GSK3326595, and GSK3368715 were synthesized and purchased from Selleck Chemicals. GSK591 was purchased from Cayman Chemical (Biomol GmbH). ARV-771 was purchased from MedChemExpress.

### Cell lines, mycoplasma contamination, and authentication.

Conventional human and murine pancreatic cancer cell lines, testing for mycoplasma contamination, and authentication are described in Supplemental Methods.

### Human primary PDAC organoid and 2D culture and pharmacotyping.

Clinical parameters for organoids are depicted in Supplemental Table 4. Establishing, culturing, and pharmacotyping of the primary PDAC models are described in Supplemental Methods.

### Epigenetic drug library and screening approach.

The epigenetic compound library was purchased from Selleck Chemicals (L1900). Three human cell lines with high MYC expression and MYC network activity (DanG, PaTu8988T, PSN1) and 3 with low MYC expression/activity (HPAC, Panc1, PaTu8988S) were used. Next, 2000 cells per well of a 96-well plate (3610, Corning Life Sciences) were seeded and treated with the drugs after 24 hours. The following dilutions were used for all drugs: 10 μM, 5 μM, 2 μM, 0.5 μM, 0.2 μM, 0.05 μM, 0.02 μM. A treatment period of 3 days was used in the screening experiment. The screening was conducted as 1 biological replicate performed as technical triplicates. ATP was measured as a surrogate for the dose-response using CellTiter-Glo assay (Promega). The AUC for each drug and cell line was determined with GraphPad Prism 5/8 (RRID: SCR_002798). The difference (ΔAUC) between the mean AUC of 3 MYC-high cell lines and 3 MYC-low cell lines was calculated, and drugs were ranked according to the *P* value of the ΔAUC. Drugs with a *P* value less than 0.05 were defined as a hit (Supplemental Table 1 and Supplemental Table 2).

### Viability assay, GI_50_ and AUC calculations, caspase-3/7 assay, clonogenic assay, flow cytometry, and drug synergy calculations.

See the Supplemental Methods for this information.

### Western blotting.

Western blotting, protein lysates, and the antibodies used are described in Supplemental Methods. Western blots were visualized using an Odyssey Infrared Imaging system (RRID: SCR_013430, LI-COR Biosciences). Protein bands were quantified using Image Studio Lite software (RRID: SCR_013715, LI-COR Biosciences). Protein expression values were normalized on expression of a housekeeping protein, and final expression values were calculated out of 3 biological replicates or 1 replicate (Figure 4, D and F).

### Quantitative PCR and RNA-Seq.

See Supplemental Methods for details.

### Proteomics of PDO lines.

See Supplemental Methods for details.

### Data sets, GSEA, STRING analysis, and loss-of-fitness scores.

Expression data sets for JNJ-64619178–treated PSN1 and DanG cells as well as HPAC-MYC-CRISPRa cells can be accessed via European Nucleotide Archive (ENA): PRJEB43040. Differentially expressed genes of JNJ-64619178–sensitive (lowest quartile) and –resistant (highest quartile) PDOs and PDCLs can be found in Supplemental Table 4. The pancreatic adenocarcinoma data set of the TCGA (RNA-Seq V2) was retrieved via the cBioPortal platform (http://www.cbioportal.org/, December 2018). Normal tissues, non-PDACs, or samples with low cellularity were excluded according to Peran et al. (28). Normalized human PDAC RNA-Seq data (ICGC) were obtained from the supplemental information of Bailey and colleagues (51). Acinar cell carcinomas and the intraductal papillary mucinous neoplasms were excluded as described recently (20). Normalized RNA-Seq data of PDCLs were obtained from the supplementary information of Brunton and colleagues (64). The mRNA expression data set of human conventional PDAC lines was downloaded via the DepMap portal (https://depmap.org/portal/) (CCLE_expression; Q3/19). Correlation data of *MYC* mRNA with *PRMT* mRNAs were downloaded via the DepMap portal (Q4/20). RNA expression profiles of murine PDAC cell lines were described recently (65). GSEA was performed with the GeneTrail3 web tool (http://genetrail.bioinf.uni-sb.de/) using default settings (66). The STRING 11.0 analysis was performed via the STRING database platform (https://string-db.org/cgi/) (67). The multiple protein pipeline with human PRMT5 and MYC was queried with default parameters (medium confidence > 0.4, no more than 5 interactors). The loss-of-fitness scores for the PDAC context of a CRISPR/Cas9 dropout screen (2) were directly retrieved via the Project Score portal (https://score.depmap.sanger.ac.uk/). Furthermore, proteomics-based expression of PRMT5 and MYC and CRISPR/Cas9 dropout gene effects were accessed via the DepMap portal (29).

### CRISPRa/CRISPRi and lentiviral transduction.

See Supplemental Methods for details.

### Data availability statement.

Expression data sets for JNJ-64619178–treated PSN1 and DanG cells as well as HPAC-MYC-CRISPRa cells can be freely accessed via ENA: PRJEB43040.

### Statistics.

All experiments were conducted in biological triplicates unless otherwise stated. In all figures the SD is depicted. Two-sided *t* test or Mann-Whitney *U* test were used to investigate statistical significance as indicated. *P* values were calculated with GraphPad Prism 5/8 (RRID: SCR_002798). For figures in which controls were normalized to 1, statistical testing was performed on non-normalized data, taking variation of controls into account. *P* values less than 0.05 were considered significant.

### Study approval.

The primary human PDAC cellular models were established and analyzed in accordance with the Declaration of Helsinki; were approved by the local ethical committee of TUM, Klinikum rechts der Isar and LMU, Klinikum der Universität München (projects 207/15, 1946/07, 330/19S, 80/17S, 5542/12, and 17-648); and written informed consent from the patients for research was obtained prior to the investigation.

## Author contributions

KL, FO, C Schneeweis, CJB, MW, MR, DS, and GS conceived and designed the study. KL, FO, C Schneeweis, ZH, HJ, LK, FB, C Schneider, AS, J Murr, RO, CW, CJB, MW, DS, MR, and GS acquired and/or analyzed and interpreted data. FO, C Schneeweis, ZH, HJ, LK, C Schneider, AS, RO, CW, GB, UMM,YX, J Mayerle, RMS, B Kuster, RR, B Kong, and C Schlag generated important models and contributed essential resources and technology. RR, MR, DS, and GS provided funding. KL, FO, C Schneeweis, ZH, LK, C Schneider, CJB, and GS drafted the manuscript. All authors revised the manuscript for important intellectual content and approved the final version submitted for publication. The order of the equally contributing first authors was determined by tossing a coin on each submission.

## Supplementary Material

Supplemental data

Supplemental table 1

Supplemental table 2

Supplemental table 3

Supplemental table 4

Supplemental table 5

## Version 1 04/19/2022

In-Press Preview

## Version 2 05/23/2022

Electronic publication
