# Supplementary material for: Epigenetic drug screening defines a PRMT5 inhibitor–sensitive pancreatic cancer subtype
Source: JCI Insight. 2022 May 23;7(10):e151353. doi: 10.1172/jci.insight.151353 (PMC9220834; doi:10.1172/jci.insight.151353)
Supplement: Supplemental data [file jciinsight-7-151353-s101.pdf]

SUPPLEMENTAL FIGURES

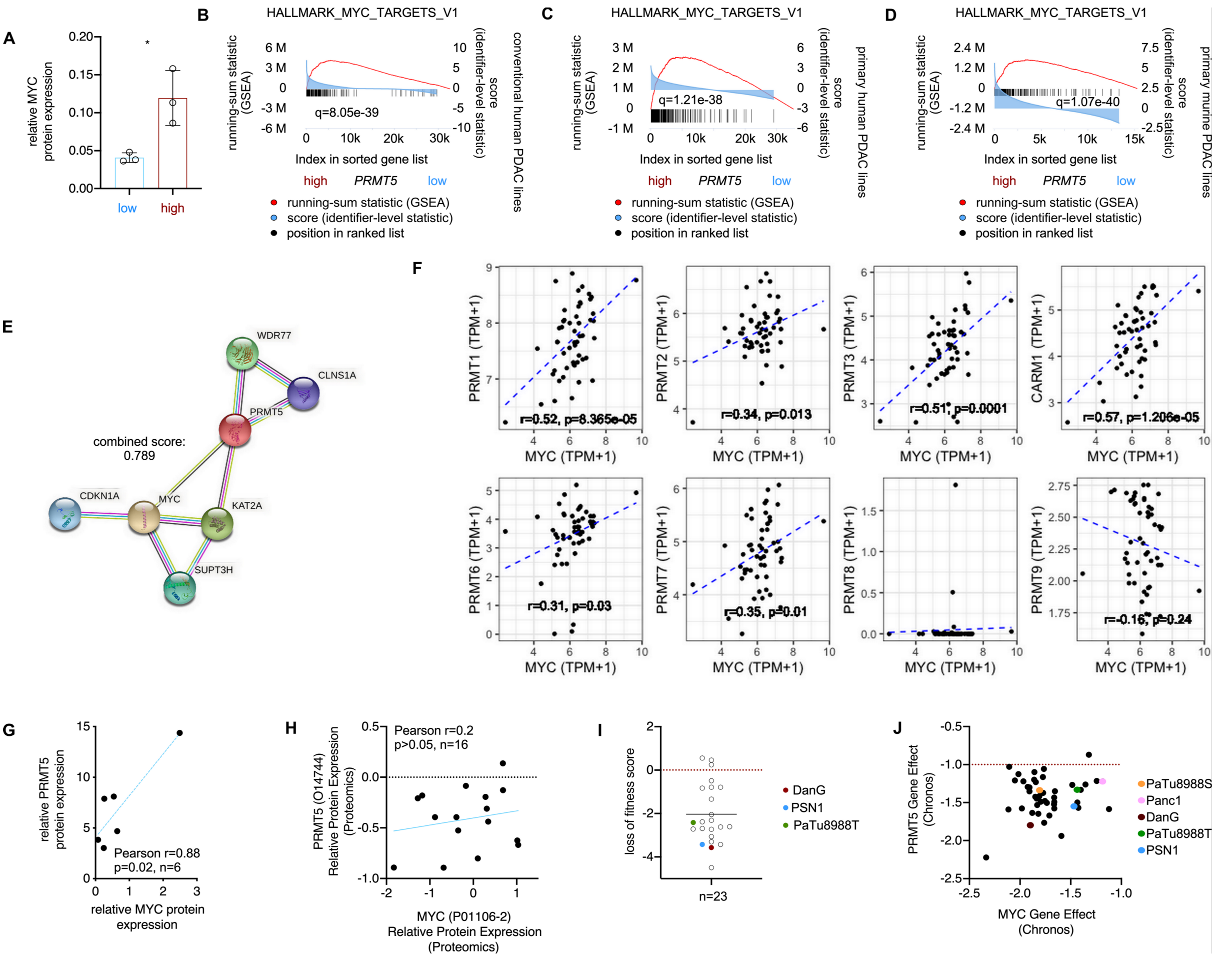

Supplemental Figure 1 *Connection of PRMT5 to MYC cross species.*

A) Quantification of MYC protein expression of the PDAC cell lines involved in the screening experiment determined by western blotting. Beta-actin was used as a loading control and MYC expression was normalized to the housekeeping gene. Results were calculated out of three independent biological replicates. *unpaired t-test: p<0.05. MYC low: Panc1, HPAC, PaTu8988S; MYC high: PSN1, DanG, PaTu8988T. **B-D)** GSEA of RNA expression datasets with high PRMT5 (expression >75th percentile) versus low PRMT5 (expression <75th percentile) mRNA expression using the Gene Trail3 web tool with B) conventional human PDAC cell lines (n=49), C) primary human PDAC cell lines (n=48), D) primary murine PDAC cell lines (n=38). Depicted is the HALLMARK signature for MYC TARGETS V1 including q values. **E)** STRING analysis of protein-protein interaction between MYC and PRMT5. A confidence score of 0.789 was calculated. **F)** Depicted is the Pearson correlation coefficient and the linear regression (in blue) between *MYC* and *PRMT1, 2, 3, 6, 7, 8, 9 and CARM1*

mRNA expression in conventional human PDAC cell lines. Data was directly retrieved from the DepMap portal and includes n=52 cell lines. The Pearson correlation coefficient r and the p values were depicted. **G-H)** Depicted is the Pearson correlation coefficient and the linear regression (in blue) between *MYC* and *PRMT5* protein expression G) determined by western blot and H) by data based on proteomics and accessed via the DepMap portal. **I-J)** Results of a CRISPR drop-out screen for the PDAC context. I) Data were retrieved from the Project Score portal. The MYC high lines included in our screening are marked, the MYC low lines were not included in the genetic screen. A negative score is statistically significant. J) Data were retrieved via the DepMap portal and a score <-1 is considered a as strong gene effect.

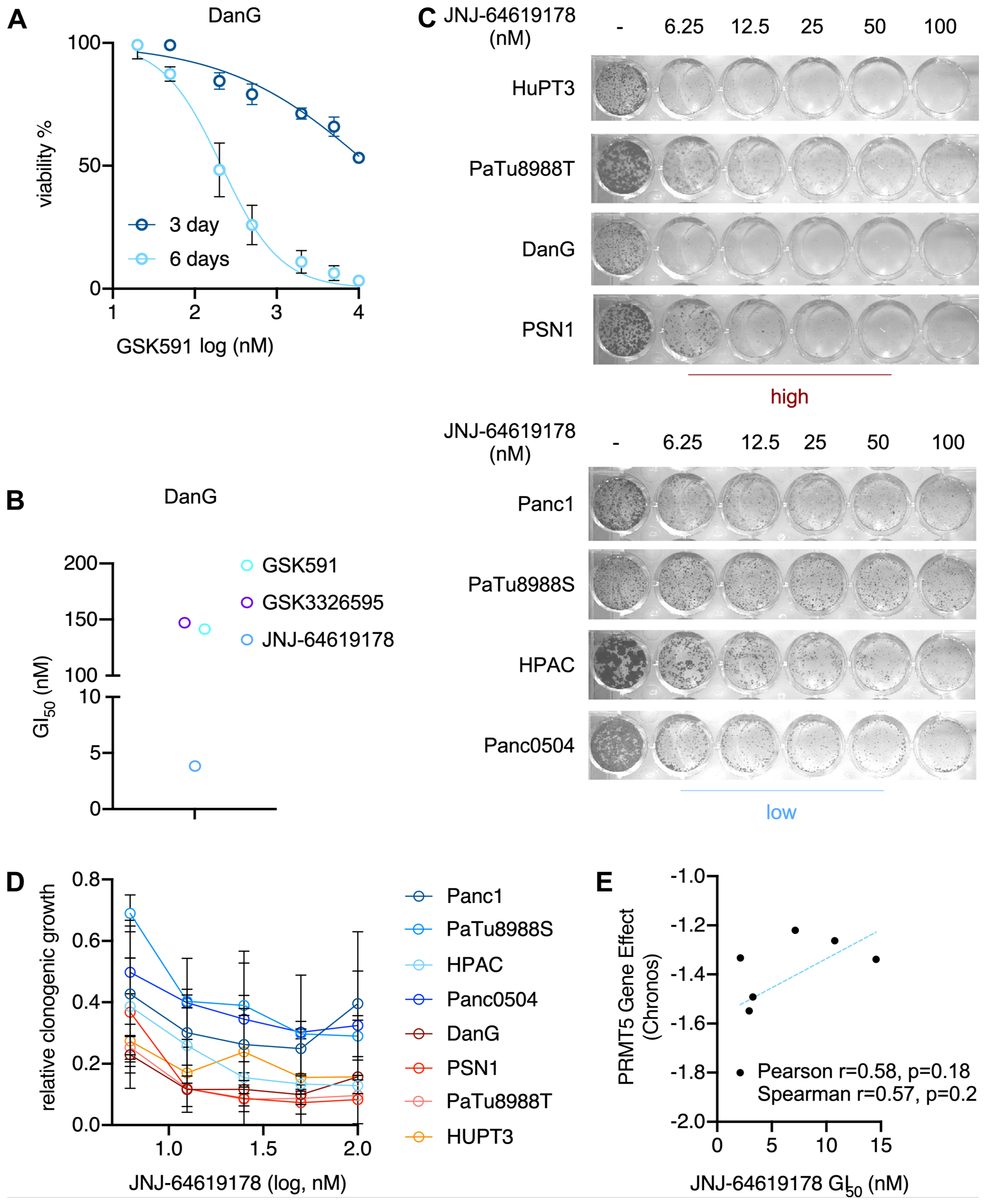

Supplemental Figure 2 *PRMT5 response over time.*

A) Viability for multi-dose treatment of DanG cells treated with the indicated doses of GSK591 over 3 or 6 days. All experiments were conducted in three independent biological replicates conducted as technical triplicates. Viability was determined using Cell Titer Glo-assays. **B)** DanG cells were treated with the indicated PRMT5 inhibitors as described in A for six days. Afterwards GI_{50} values were calculated and are depicted. **C)** Indicated PDAC cell lines were treated with JNJ-64619178 and clonogenic growth was monitored. One representative assay is depicted. **D)** Clonogenic growth of the indicated cell lines of C). Shown is the mean +/- SD

of three independent biological replicates. **E)** JNJ-64619178 GI_{50} values, based on the clonogenic growth curves of C) and D), were correlated with the gene effects of the PRMT5 CRISPR-drop out screens. Gene effects were retrieved from the DepMap portal.

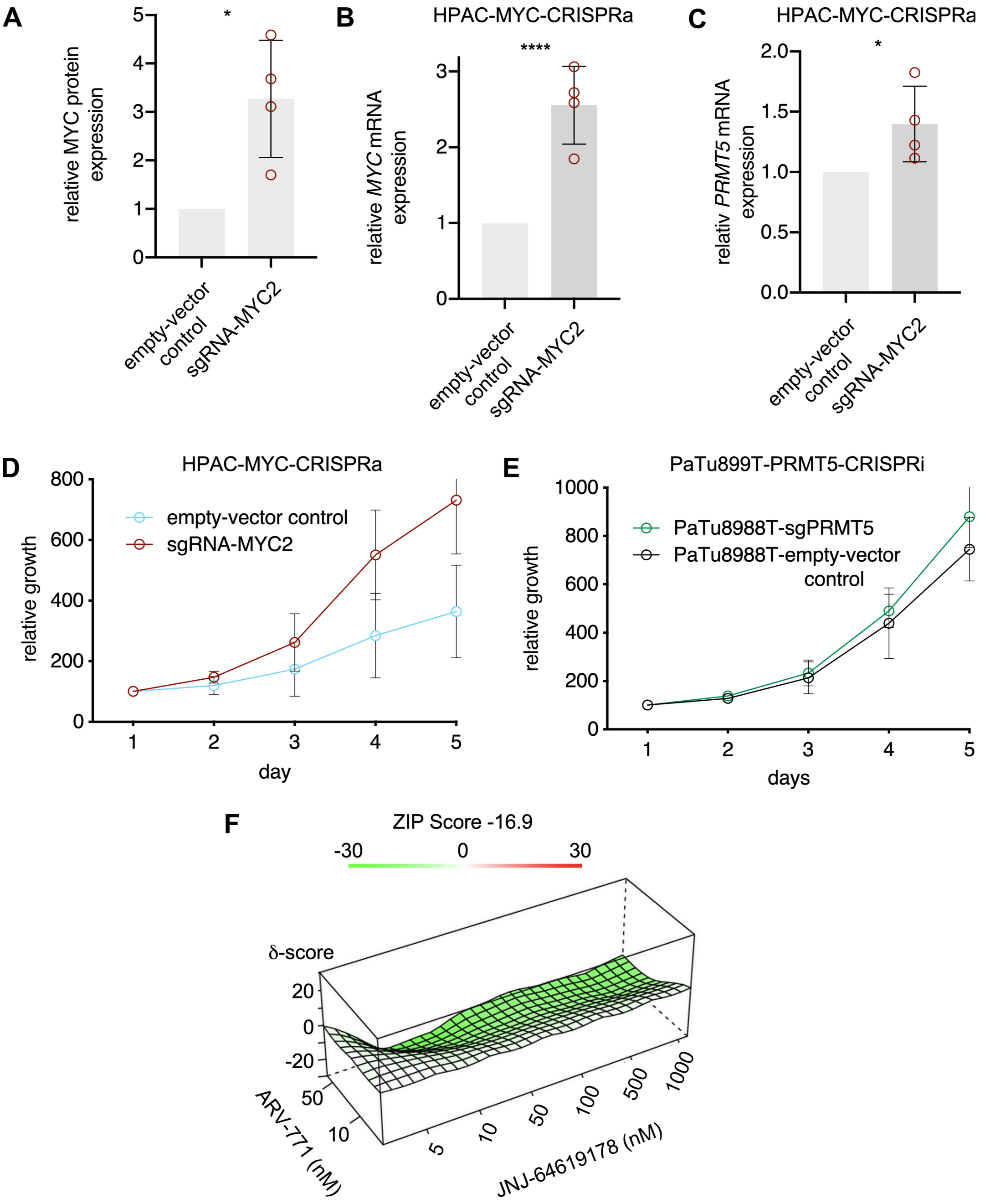

Supplemental Figure 3 *Models to manipulate MYC and PRMT5 expression.*

A) Quantification of MYC protein expression of MYC-CRISPRa HPAC cells. Protein expression was determined out of four biological replicates via western blot. * p value of an unpaired t-test <0.05. n=4. **B-C)** Quantification of B) *MYC* and C) *PRMT5* mRNA expression of MYC-CRISPRa HPAC cells. mRNA expression was determined out of four biological replicates performed as technical triplicates by qPCR. *GAPDH* was used to normalize the expression. *p value of an unpaired t-test <0.05, ****p value of an unpaired t-test <0.0001. **D-**

E) Growth curves of D) HPAC-MYC-CRISPRa (n=2) and E) PaTu8988T-PRMT5-CRISPRi (n=2) cell lines. **F)** Synergy map of JNJ-64619178 and ARV-771 co-treated DanG cells.

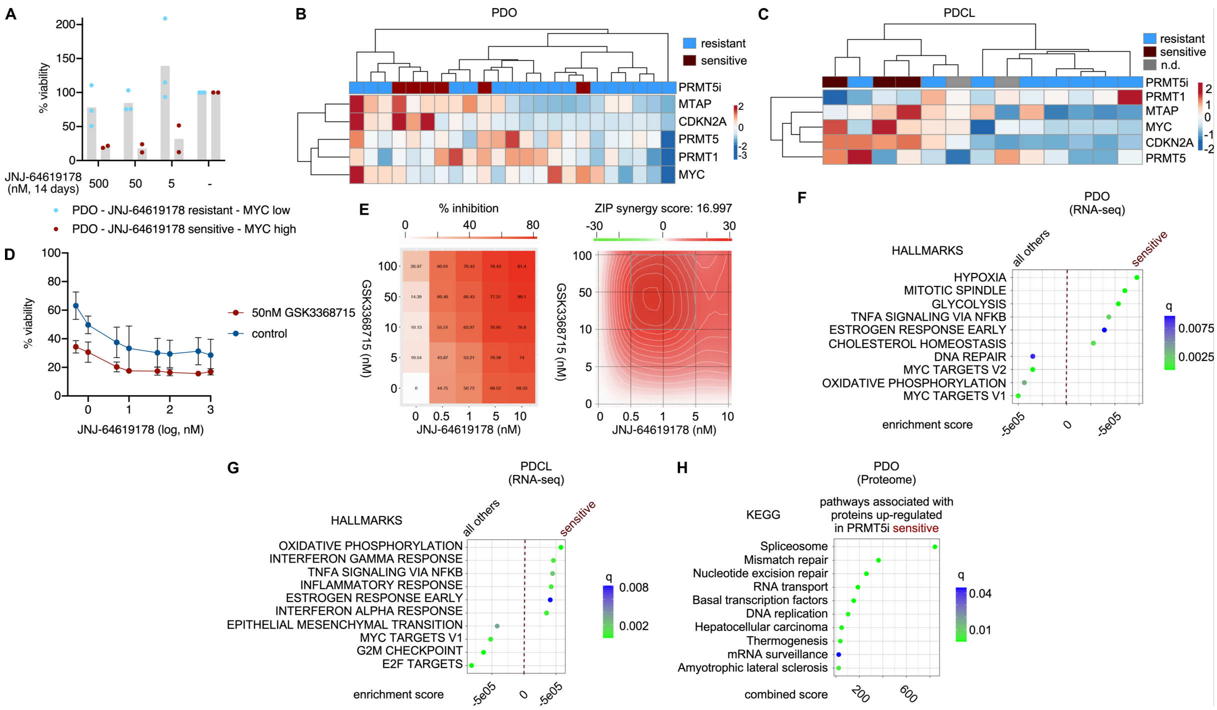

Supplemental Figure 4 *Long term PRMT5i treatment and combination with PRMT1i.*

A) Viability measurement of a MYC high and a MYC low organoid lines after 14 days of treatment with JNJ-64619178, determined by CellTiterGlo. In the MYC high PDO, the experiment was performed with two independent biological replicates, in the MYC low PDO line, the experiment was performed with three independent biological replicates. **B)** mRNA expression based on a RNA-seq of the investigated organoids (n=23, for one PDO line included in the sensitivity testing, no RNA-seq was available). Expression of the indicated mRNAs is illustrated by a heatmap. Subgroup is indicated as blue (resistant) and red (sensitive). **C)** mRNA expression based on a RNA-seq of investigated PDCL (n=13). Expression of the indicated mRNAs is illustrated by a heatmap. Subgroup is indicated as blue (resistant) and red (sensitive). Grey: JNJ-64619178 sensitivity not determined (n.d.). **D)** Viability for multi-dose treatment of HUPT3 cells treated with the indicated doses JNJ-64619178 in combination with DMSO control or 50nM GSK3368715 over 6 days. All experiments were conducted in three independent biological replicates conducted as technical triplicates. Viability was determined using Cell Titer Glo-assays. **E)** Synergy map and Synergy score of JNJ-64619178 and GSK3368715 co-treated HUPT3 cells. **F)** JNJ-64619178 GI_{50} values of PDOs were grouped in quartiles and differential expressed genes of most sensitive

(1st quartile, n=6) and all other PDOs (2-4th quartile, n=17) were calculated. The log fold-change was used as a rank to perform a pre-ranked GSEA using the Gene Trail3 web tool. Depicted are the top ten HALLMARK signatures ranked by enrichment score values. The enrichment score is depicted and the q value is color coded. **G)** JNJ-64619178 GI_{50} values of PDCL were grouped in quartiles and analyzed corresponding to F). Sensitive (1st quartile, n=3), Resistant (1st quartile, n=8). Depicted are the top ten HALLMARK signatures ranked by enrichment score values. The enrichment score is depicted and the q value is color coded. **H)** Proteomics based protein expression of PRMT5i sensitive (1st quartile, n=6) and resistant (4th quartile, n=6) PDOs was used to determine differentially expressed proteins. All proteins up-regulated in sensitive PDOs were analyzed using the Enrichr web tool. Depicted are the KEGG signatures with an adjusted p-value: q <0.05. The q-value is color coded and the combined score is depicted.

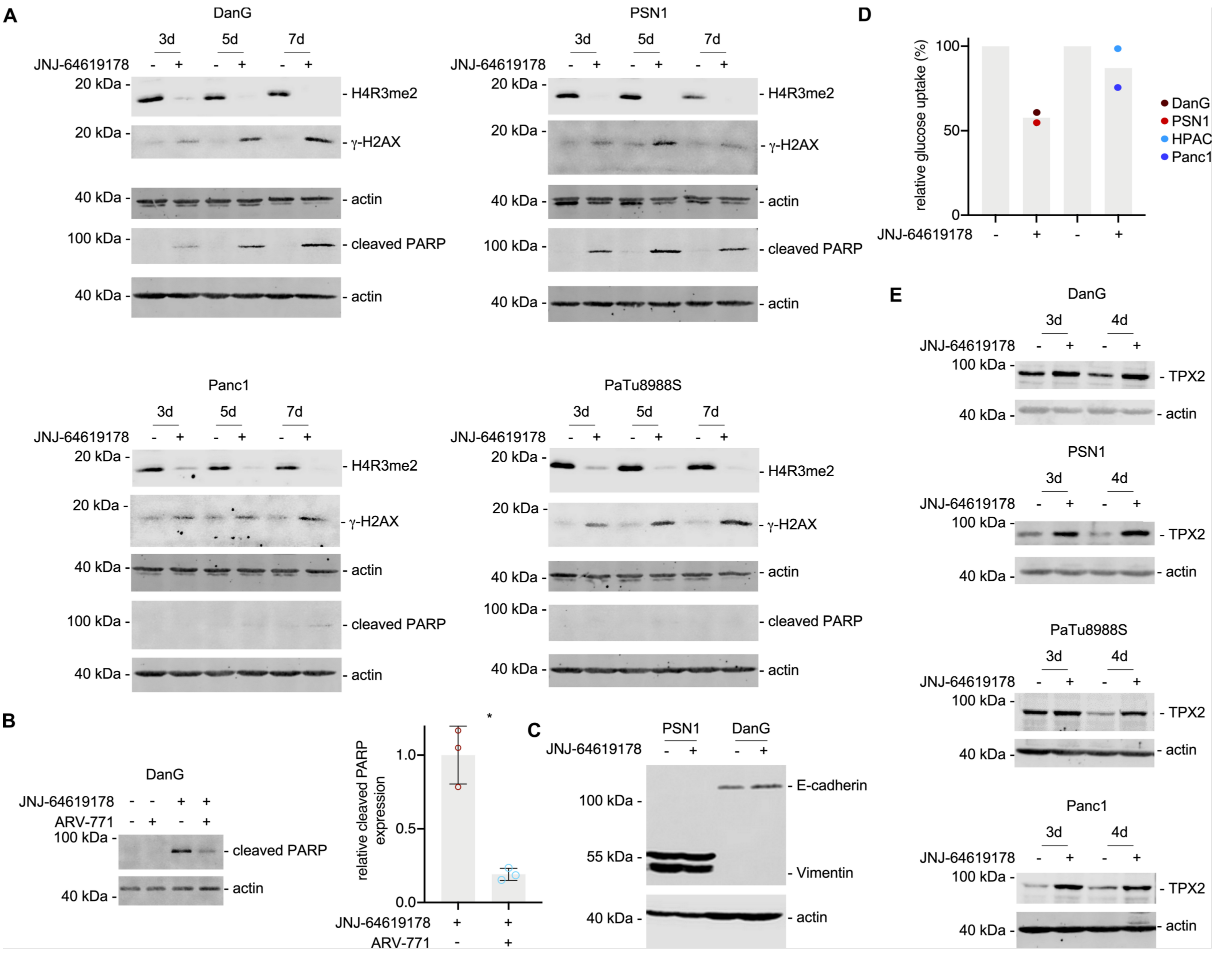

Supplemental Figure 5 *Cellular JNJ-64619178 response.*

A) Western blot analysis of indicated proteins in two MYC high (DanG, PSN1) and two MYC low (Panc1, PaTu8988S) cell lines with and without 20nM JNJ-64619178 treatment after indicated time points. Beta-actin served as a loading control, DMSO was used as control treatment. One representative experiment out of three biological replicates is depicted. **B)** DanG cells were treated with 20 nM JNJ-64619178, 20 nM ARV-771, the combination thereof, or were left as vehicle treated controls for 96 hours. Left panel: PARP western blot. Beta-actin served as a loading control. Right panel: quantification of three independent experiments. * paired t-test $p<0.05$. **C)** E-cadherin and Vimentin western blot analysis of indicated cell lines which were treated with 20 nM JNJ-64619178 for 3 days. Beta-actin served as a loading control. **D)** Glucose uptake assay of two MYC high (DanG and PSN1) and two MYC low (HPAC and Panc1) cell lines after 3 days treatment with 20 nM JNJ-64619178. Bars represent the glucose uptake of the MYC low and MYC high groups relative to the control lines without

treatment, which were arbitrarily set to 100%. Experiment was performed as three biological replicates in each line. **E)** TPX2 western blot analysis of indicated cell lines treated for three and four days with 20nM JNJ-64619178 or were left as a vehicle treated control. Beta-actin served as a loading control. One representative experiment out of two replicates is depicted.

Supplementary Methods

Cell lines, Authentication

Human pancreatic cancer cell lines were cultured in Dulbecco's Modified Eagle's Medium - high glucose (#D5796, Sigma-Aldrich, Darmstadt, Germany) or RPMI GlutaMAX® (#61870036, Life Technologies, Darmstadt, Germany) medium with 10% (v/v) fetal calf serum (FCS) (#TMS-013-B, Merck Millipore, Berlin, Germany) and 1% (v/v) Penicillin/Streptomycin (#15140122, Life technologies, Darmstadt, Germany), depending on the cell line (DMEM: Panc1 (RRID:CVCL_0480), PaTu8988T (RRID:CVCL_1847), PaTu8988S (RRID: CVCL_1846); RPMI: DanG (RRID: CVCL_0243), HPAC (RRID:CVCL_3517), PSN1 (RRID:CVCL_1644), IMIM-PC1 (RRID: CVCL_4061), Panc0504 (RRID: RRID:CVCL_1637), HUPT3 (RRID: CVCL_1299)). They were authenticated regularly (10/2021) by Multiplexion (Multiplexion GmbH, Heidelberg, Germany) or Microsynth (Microsynth AG, Balgach, Switzerland). All murine pancreatic cancer cell lines were established from $Kras^{G12D}$-driven mouse models of pancreatic cancer (1). Identity of murine cell lines was verified by genotyping PCR. Murine cell lines were cultured in high glucose DMEM medium (#D5796, Sigma-Aldrich, Darmstadt, Germany) with 10% (v/v) FCS (Merck Millipore, Berlin, Germany) and 1% (v/v) Penicillin/Streptomycin (Life technologies, Darmstadt, Germany). For splitting, all cell lines were washed with Dulbecco's phosphate buffered saline (PBS) (Sigma-Aldrich, Darmstadt, Germany) and detached using EDTA (Versen), 1% (in PBS w/o Ca^{2+} w/o Mg^{2+})(#L2113, Biochrom, Darmstadt, Germany) diluted 1:20 in PBS. The murine cell line PPT-9091 was transduced with the pBabepuro-myc-ER construct, which was a gift from Wafik El-Deiry (Addgene plasmid # 19128, RRID:Addgene_19128). Transduction procedure and the line is described (2, 3). $IMIM\text{-}PC1^{MYC\text{-}ER}$ cells were described recently (2).

Mycoplasma Contamination Test

Screen for mycoplasma contamination of all cell lines was done regularly by PCR. Briefly, cells were seeded in 6-well plates in 3ml of their respective medium without antibiotics for 1 week. 2ml of the medium was taken and centrifuged at RT at 250xg for 2 minutes. The supernatant was pipetted into a fresh tube and centrifuged again for 10 minutes at RT at 20000xg. The

supernatant was removed, the pellet was resuspended in the remaining liquid and heat inactivated at 95°C for 3minutes. 2µl was used as a template in the following PCR (PCR mix (1x):15µl Red-Taq Premix (Sigma-Aldrich), 2µl 5`Primer dilution (10µl of each 5`Primer (10µM) + 30µl H2O), 2µl 3`Primer dilution (10µl of each 3`Primer (10µM) + 70µl H2O), 9µl H2O). The following primers were used: 5`Primer 1: 5`C G C C T G A G T A G T A C G T T C G C 3`; 5`Primer 2: 5`C G C C T G A G T A G T A C G T A C G C 3`; 5`Primer 3: 5` T G C C T G G G T A G T A C A T T C G C 3`; 5`Primer 4: 5` T G C C T G A G T A G T A C A T T C G C 3`; 5`Primer 5: 5`C G C C T G A G T A G T A T G C T C G C 3`; 5`Primer 6: 5`C A C C T G A G T A G T A T G C T C G C 3`; 5`Primer 7: 5`C G C C T G G G T A G T A C A T T C G C 3`; 3`Primer 1: 5`G C G G T G T G T A C A A G A C C C G A 3`; 3`Primer 2: 5`G C G G T G T G T A C A A A A C C C G A 3`; 3`Primer 3: 5`G C G G T G T G T A C A A A C C C C G A 3`. The following PCR protocol was used: 95°C 15 minutes; (94°C 1 minute, 60°C 1 minute, 74°C 1 minute) x40; 72°C 10 minutes. The PCR product was loaded on a 2% agarose gel, separated via gel electrophoresis for 1 hour at 100V and visualized using UVsolo TS Imaging System (Biometra, Analytik Jena AG, Jena, Germany).

Human primary PDAC Organoid and 2D Culture

The primary human PDAC 3D organoid and 2D models were established and analyzed in accordance with the declaration of Helsinki, were approved by the local ethical committee TUM, Klinikum rechts der Isar and LMU, Klinikum der Universität München (Project 207/15, 1946/07, 330/19S, 80/17S, 5542/12, and 17-648), and written informed consent from the patients for research use was obtained prior to the investigation. Clinical parameters of the 3D lines are depicted in Table S4.

Generation of Organoids

Primary patient-derived PDAC 3D organoids were generated from fine needle aspiration/biopsies (FNA/B) or surgical resected cancers. The isolation process was conducted shortly (<20 minutes) after receiving the sample on ice. After washing the sample with PBS and a centrifugation step (5 minutes, 1000 rpm, 4°C), the supernatant was aspirated, and the sample was transferred to a cell culture plastic dish to mechanical dissect the

specimen with a scalpel into small pieces. FNA/B samples were transferred into a 15-ml falcon with 5 ml PBS; surgical samples were transferred into a 15-ml Falcon tube filled with 5 ml of digestion buffer (Advanced DMEM-F12 (#12634010 Thermo Fisher Scientific, Massachusetts, USA), 1 M Hepes, 1 x Glutamax Supplement (#35050061, Thermo Fisher Scientific, Massachusetts, USA), 1 x Primocin (#ant-pm-2, Invivogen), 6 mg/ml Collagenase II (#17101-015 Thermo Fisher), 2.5% Fetal Calf Serum (#TMS-013-B, Merck-Millipore, Berlin, Germany). The sample was incubated for 1h in a Rotating Mixer with 30 rpm. Next, the falcon was placed upright for 10 seconds to allow sedimentation of debris. The supernatant was then transferred in a new 15-ml falcon tube. FNA/Bs and surgical specimen were then centrifuged for 5 minutes at 1000 rpm and 4°C. The supernatant was discarded, and the pellet resuspended in 3 ml red blood cell lysis buffer (#A1049201, ACK Lysis Buffer from Thermo Fisher Scientific, Massachusetts, USA) for 3 to 10 minutes at room temperature. After a washing step in PBS and centrifugation (5 minutes, 1000 rpm, 4°C), the samples were digested in 3 ml TrypLE (#12604039, Thermo Fisher Scientific, Massachusetts, USA) for 3 - 5 minutes at 37°C. After an additional washing and centrifugation step, the cell pellet was dissolved in Matrigel (#354230, Corning Life Sciences, Corning, NY 14831 USA) and plated on a prewarmed 24-well plate (depending on the sample size 2 – 8 wells on a 24 well plate with 50 µl Matrigel/well). After 10 minutes, 500 µl of PDO culture medium (see below) was added to each well.

Passaging of the organoids

The 24-well plate with the organoids was removed from the incubator and the media was aspirated. Then, 250 µl of Cell Recovery Solution (#11543560, Fisher Scientific) was added to each well. Subsequently, the mixture of Matrigel, organoids and Cell Recovery Solution was resuspended with 1 ml of ice-cold PBS w/o Ca^{2+} w/o Mg^{2+} (Sigma Life Sciences), supplemented with 0.1% BSA (#11930, Serva, Heidelberg, Germany), transferred into a 15-ml falcon tube and centrifuged at 1000 rpm at 4°C for 5 minutes. Afterwards, the supernatant was aspirated and the organoid cell pellet was resuspended in 2 ml PBS. Then the falcon tube was filled with 6 ml cold PBS and centrifuged at 1000 rpm at 4°C for 5 minutes. The supernatant was aspirated and the cell pellet resuspended with 50 µl/well of Matrigel. The

appropriate number of cells per wells for subcultivation was dependent on the growth rate and ranges from a splitting ratio of 1:1 till 1:3. The medium was changed twice a week.

Detection of KRAS mutations

We only used organoids with mutated *KRAS*. Mutations in the oncogene (see Table S4) were determined by Sanger Sequencing. In brief, organoid DNA was isolated via the DNA/RNA Micro Kit (#80284, Qiagen, Germany) and amplified via PCR using the Q5® High-Fidelity DNA Polymerase (#M0491S, New England Biolab) with the following primers hKRAS_ex2_flank_Fw: 5`G G T A C T G G T G G A G T A T T T G A T A G T G 3` and hKRAS_ex2_flank_Rv 5`G G T C C T G C A C C A G T A A T A T G C A 3`. Sanger sequencing was done by Eurofins (Ebersberg, Germany).

Pharmacotyping of Organoids

Only organoids were analyzed which re-grow after a freeze thaw cycle. Pharmacotyping of organoids was conducted in between passage 5-17. Organoids were cultured in 24-well plates embedded in 50 µl of Matrigel and 500 µl of PDO culture media (DMEM-F12 (#11320033, Thermo Fisher), 5mg/ml D-Glucose (#G8270, Sigma-Aldrich), 0.5% ITS Premix (#354350, Fisher Scientific), 5 nM 3,3,5-Triiodo-L-Thyronine (#T0821, Sigma Aldrich), 1 µM Dexamethason (#D1756, Sigma Aldrich), 100 ng/ml Cholera Toxin (#C9903, Sigma Aldrich), 1% Pen-Strep (Thermo Fisher), 5% NU-Serum IV (#355500, Fisher Scientific), 25 µg/ml Bovine Pituitary Extract (#P1167, Sigma Aldrich), 10 mM Nicotinamide (#N3376, Sigma Aldrich), 100 µg/ml Primocin (#ant-pm05, Invivogen), 0.5 µm A83-01 (#2939, Tocris), 10% RSPO1-conditioned medium (R-spondin-1 overexpressing cell line HEK293T, provided by the Hubrecht Institute (Uppsalalaan 8, 3584 CT Utrecht, Netherlands), 100 ng/ml Recombinant Human Heregulin-1 (#100-03, Peprotech)). For pharmacotyping, the 24-well plate with the organoids was removed from the incubator and the media was aspirated. Then, 250 µl of Cell Recovery Solution ((#11543560, Fisher Scientific) was added to each well. Subsequently, the mixture of Matrigel, organoids and Cell Recovery Solution was resuspended with 1 ml of ice-cold PBS w/o Ca^{2+} w/o Mg^{2+} (Sigma Life Sciences), supplemented with 0.1% BSA (Serva, Heidelberg, Germany), transferred into a 15-ml falcon tube and centrifuged at 1000 rpm at 4°C

for 5 minutes. Afterwards, the supernatant was aspirated and the organoid cell pellet was resuspended in 2 ml PBS. Then the falcon tube was filled with 6 ml cold PBS and centrifuged at 1000 rpm at 4°C for 5 minutes. The supernatant was removed completely, and the cell pellet resuspended with 1 ml of TrypLE and incubated at 37°C for 1 min. Afterwards, 7 ml of warm DMEM-F12 was added and the cells were centrifuged at 1000 rpm at 4°C for 5 minutes. The supernatant was removed completely, and the cell pellet was resuspended in 1 ml of warm PDO culture Media supplemented with 10 µM Rho Kinase Inhibitor (#TB1254-GMP, Y-27632, Tocris).

The cells were counted in a disposable Hemocytometer (#MDH-2N1-50PK, Merck, Germany) and 500 cells/well in 20 µL drug screen solution (2 µL Matrigel + 18 µL PDO growth medium + 10 µM Y-27632) were transferred to a 384-well plate (#CLS3765, Corning Life Sciences). To prevent evaporation 50 µL of PBS were filled to empty wells on the 384-well plate followed by centrifugation of the plate (10 seconds at 500 rpm). To incubate the cells, the plate was placed at 37°C and 5% CO_2. After 24 hours the drugs were added in 4 µl of PDO Medium. After a treatment period of six days, 5 µl CellTiter-Glo® Luminescent Cell Viability reagent (#G7573, Promega, Fitchburg, Wisconsin, USA) was added to each well. After 10 minutes of gentle shaking at 300 rpm and 20 minutes of incubation at room temperature, luminescence was measured on a FLUOstar OPTIMA microplate reader (BMG Labtech GmbH).

Long-term treatment organoids

For the long-term treatment, organoids were seeded in a 50 µl Matrigel dome in one well per concentration of a 24-well plate. The cell number was tested for each line individually, due to the long growth period in the well (25.000 – 50.000 cells per well, depending on the line). The next day, JNJ-64619178 or DMSO as a control was added in 100 µl PDO-medium. The medium was changed after additional 6 days and drugs were added freshly on the same day. After 14 days, 125 µl CellTiter-Glo® was added to each well. After 15 minutes of shaking at 450 rpm and 20 minutes of incubation at room temperature, the supernatant was transferred into wells of a clear-bottom 96-well plate (#3610, Corning Life Sciences, New York, USA).

Luminescence was measured on a FLUOstar OPTIMA microplate reader (BMG Labtech GmbH).

Proteomics of PDO lines

Protein digestion and peptide desalting.

The cells were washed with PBS twice and then the lysis buffer which contained 2% SDS and 40 mMTris-HCl was used to lysate these cells. After that, the cell lysate was heated up to 95oC for 5 min. Then, TFA was added to the cell lysate with a final concentration of 1%. After incubation for 2 min, 40% NMM solution was added to adjust the pH value to about 7.5. The protein concentration was measured by BCA method later.

Protein digestion was performed following the single-pot solid-phase-enhanced sample preparation (SP3) protocol (4). Disulfide bridges were reduced by adding dithiothreitol (DTT) and cysteines were alkylated with chloroacetamide (CAA) for 0.5h at 37 °C in the dark. Finally, trypsin (Roche) was added to the digestion buffer at an enzyme:protein ratio of 3:100 (w/w), and the digestion was performed overnight at 37 °C in a ThermoMixer at 1,000 rpm. After digestion, FA was added with a final concentration of 1%. The samples were desalted using the Oasis HLB 96-well µElution Plate (Waters, 30 µm). Peptides were eluted from HLB plate with 70% ACN, 0.1% FA solution. The elutes were frozen and dried in a SpeedVac centrifuge.

Off-line peptides fractionation

The AssayMap Bravo platform (Agilent technologies) was used to do the basic reverse phase fractionation, with the reverse phase (RP-S) cartridges. The cartridges were primed sequentially with 150ul isopropanol, ACN and solvent B (80% ACN, 10 mM NH4FA in water, pH=10.0). The flow rate is 50µl/min. After that, the cartridges were washed with 100 µl of solvent A (25 mM NH4FA in water, pH=10.0) with a flow rate of 10 µl/min. The peptides were dissolved in 100 µl of solvent A, and loaded onto the cartridges with a flow rate of 5µl/min, the follow-through (FT) was collected. The cartridges were washed with 50 µl of solvent A, the washing solution was collected in the flow-through. The peptide was eluted with 25 mM NH4FA (pH = 10.0) and had a gradual increase in ACN (5%, 10%, 15%, 20%, 25%, 30%, 80%). Finally,

we combined seven fractions with flow-through into four fractions (5% + 25%, 10% + 30%, 15% + 80%, 20% + FT).

LC–MS/MS analysis

The Microflow LC-MS/MS system was set up with a modified vanish pump combined with a Q Exactive HF-X (Thermo Fisher Scientific) mass spectrometer constructed in the previous report (5). The peptides were dissolved in 20 µl of 0.1% FA solution, and 15 µl of the sample was injected directly onto an Acclaim PepMap100 C18 column (Thermo Fisher Scientific, 2 µm, 1 mm ID x 150 mm, Cat. No. 164711) using solvent A (0.1% FA in water, 3% DMSO). The samples were separated using a linear gradient of 3%-28% solvent B (0.1% FA, 3% DMSO in acetonitrile) at a flow rate of 50 µl/min during 60 minutes.

Data processing and analysis

The raw MS data files were processed with MaxQuant v1.6.2.34 and searched against the human protein database, which contains 20,230 entries (downloaded 06/2017) .The default MaxQuant parameters were used. Label-free quantification and match-between-run matching function was enabled. The result were further processed with Perseus v1.6.15.0. The intensity of all protein groups was upload to perseus. The reverse proteins and potential contaminates were removed. Two samples t-test was performed to compare different drug sensitive cell line (lowest quartile (n=6) versus highest quartile (n=6). Differentially expressed proteins are depicted in Table S4. All upregulated proteins (n=369) were analyzed with the Enrichr web tool (6).

Human primary-dispersed PDAC 2D lines

Generation and propagation of human primary 2D cell lines was described (7). The lines B211, B290, huPDAC11, huPDAC3, huPDAC7, huPDAC17, PDC40, PDC49, PDC56, PDC117, PDC148, and huPDAC1 were cultured in RPMI GlutaMAX® (#61870036, Life Technologies, Darmstadt, Germany) supplemented with 20% FCS and 1% Penicillin-Streptomycin (Merck, Sigma-Aldrich, Darmstadt, Germany). The lines PACADD-119, PACADD-135, PACADD-137, PACADD-159, PACADD-161, and PACADD-165 were purchased from the Leibniz Institute DSMZ - German Collection of Microorganisms and Cell Cultures GmbH

(Braunschweig, Germany) and were cultured in Advanced DMEM (#12491023, Life Technologies, Darmstadt, Germany). The cells were used in-between passage 13-40 in all experiments.

Viability assay

500 to 1,000 cells per well for 6 day treatments or 2,000 cells per well for 3 day treatments, were seeded in 100µl medium in white, clear-bottom 96-well plates (#3610, Corning Life Sciences, New York, USA). Drugs were added in 20µl medium the next day and after 3 or 6 days incubation time, the plate was put out of the incubator to equilibrate to RT. 25µl CellTiter-Glo® reagent (Promega, #G7573) (buffer and substrate mixed 1:1) from Promega (Fitchburg, Wisconsin, USA) was added to each well. After 10 minutes of gentle shaking at 300 rpm and 20 minutes of incubation at RT protected from light, luminescence was measured on a FLUOstar OPTIMA microplate reader with a gain of 1500 (BMG Labtech GmbH, Ortenberg, Germany).

GI_{50} and AUC calculations, Synergy ZIP-score calculation

The half-maximal growth inhibitory (GI_{50}) concentration values (non-linear regression model) and the area-under-the-curve (AUC) were calculated with GraphPad Prism 5 (RRID:SCR_002798, GraphPad Software, California, USA). To determine synergism between drugs, the Synergyfinder platform was used (https://synergyfinder.fimm.fi/synergy) (8). Via this platform, a Zero interaction potency (ZIP) model was applied to a dose-response matrix using default settings (9).

Caspase 3/7 assay

The cells were seeded and treated like described for the cell viability assays in technical triplicates on white, clear-bottom 96-well plates (#3610, Corning Life Sciences, New York, USA). At the indicated time points, the Caspase-Glo® 3/7 Assay System (#G8090) from Promega (Fitchburg, Wisconsin, USA) was used to determine executioner caspase activity. Briefly, 70µl of liquid were taken from each well and discarded. Buffer and substrate solutions were mixed and 50µl of this solution was pipetted into each well, resulting in a 1:1 ratio of medium and solution. The plates were shaken for 30 sec at 300 rpm and incubated for 30

minutes at RT protected from light. Finally, luminescence was measured on a FLUOstar OPTIMA microplate reader with a gain of 1500 (BMG Labtech GmbH, Ortenberg, Germany). Results were calculated over three biological replicates conducted as technical triplicates.

Clonogenic assay

1,000 – 4,000 cells were seeded in 500µl medium in 24-well plates, depending on the cell line (1,000 for PSN1, PaTu8988T and PPT9091^{MYCER}, 4,000 for PaTu8988S and Panc0504, 3,000 for all others). After 24 hours, drugs in different concentrations were added in 50µl of medium followed by culturing for one week. Afterwards the medium was removed, and cells were washed with PBS. The cell colonies were stained by adding 250µl Crystal Violet solution (2.5% (v/v) EtOH and 4% (w/v) Crystal Violet (Sigma-Aldrich, Darmstadt, Germany) in H_2O) to each well and incubating the colonies for 30 minutes on a shaker at RT. Then the wells were washed 3 times with tap water, dried at least overnight and subsequently visualized using a flatbed scanner (Seiko Epson K.K., Suwa, Nagano, Japan). For quantification 600µl of 1% (w/v) sodium dodecyl sulfate (SDS) (Serva Electrophoresis GmbH, Heidelberg, Germany) solved in H_2O were added to each well and the plates were shaken over night at room temperature. Absorbance of the dissolved Crystal Violet was measured at 595nm on a CLARIOstar microplate reader (BMG Labtech GmbH, Ortenberg, Germany). Values were normalized on each control and means were calculated out of 3 biological replicates.

Flow cytometry

Cells were seeded in 5ml of their respective medium in a 10cm cell culture dish, treated the next day and harvested at the indicated time points. Cells were detached by trypsination (Trypsin – EDTA Solution 10x from Sigma, Darmstadt, Germany) and added to their supernatant. Cells were centrifuged at 1000xg for 5 minutes at 4°C, the supernatant was removed and the pellet was resuspended in 1ml 70% Ethanol (-20°C) and stored stored at 4°C. At the time of analysis, 1ml ice cold PBS was added and after centrifugation at 1000xg for 5 minutes at 4°C the pellet was resuspended in 1ml PBS. 2.5µl of RNase (stock concentration 20mg/ml; Fermentas GmbH, St. Leon-Rot, Germany) were added and the samples were incubated for 60 minutes at 37°C protected from light. For cell cycle analysis

50µl of propidium iodide (PI) (stock concentration 2mg/ml; Sigma, Darmstadt, Germany) were added, samples were incubated for at least 5 minutes, filtrated and measured at the Gallios flow cytometer (Beckman Coulter, Brea, California, USA). Results were analyzed with FlowJo™ Software (RRID:SCR_008520, FlowJo, LLC, Ashland, Oregon, USA) and three biological replicates were analyzed.

Western blotting

For determination of basal protein expression, cells were harvested at 80% confluency. For preparing the whole cell lysates, dishes were put on ice, medium was removed and cells were washed three times with icecold PBS. After removal of PBS, 100µl of lysis buffer (# 9803, Cell Signaling Technology, Danvers, Massachusetts, USA) supplemented with protease inhibitor and phosphatase inhibitor (#11873580001, Protease inhibitor cocktail complete EDTA free, Roche Diagnostics, Mannheim, Germany and Phosphatase-Inhibitor-Mix I, #39050, Serva Electrophoresis GmbH, Heidelberg, Germany) was pipetted to the dishes, cells were collected using a cell scraper, lysed by incubation for ten minutes on ice, and subsequently frozen at -80°C. For determination of apoptosis markers (cleaved PARP western blots), supernatant was included. Protein concentration was analyzed by Bradford Assay (Bradford Reagent, Serva Electrophoresis GmbH, Heidelberg, Germany). Bradford reagent was diluted 1:5 with H_2O and 300µl were pipetted into each well of a clear 96-well plate. As a standard dilution series, Bovine Serum Albumin (1µg/µl; #11930.03, fraction V, Serva Electrophoresis GmbH, Heidelberg, Germany), was used. Afterwards, absorbance was measured on a Multiskan FC microplate reader (Thermo Fisher Scientific, Massachusetts, USA) at 595nm and protein concentrations were calculated using the standard curve and the mean of the triplicates. Samples were normalized and diluted accordingly in protein loading buffer (45.6 mM Tris-HCl pH 6.8, 2% SDS, 10% glycerol, 1% β-mercaptoethanol, 0.01% bromophenol blue) and heated for 5 minutes at 95°C. Protein samples were loaded to 10% (cleaved PARP, MYC, TPX2, and PRMT5) or 15% (γH2A.X and H4R3me2sy) polyacrylamide gels with a marker (prestained protein ladder PageRuler™ from Thermo Fisher Scientific, Inc., Waltham, Massachusetts, USA) and separated using SDS-page gel electrophoresis at 80 – 100V for 2 – 3 hours in

running buffer (25 mM Tris, 192 mM Glycine, 0.1% SDS in H_2O). Proteins were transferred to a nitrocellulose membrane (Merck-Millipore, Berlin, Germany) using a wet blot system (Bio-Rad Laboratories Inc., Hercules, California, USA) with transfer buffer (25 mM Tris, 192 mM Glycine, 20% Methanol in H2O adjusted to pH 8.3) at 350mA for 2 hours. The membranes were blocked for 45min in 5% (w/v) skim milk (#T145.3, Carl Roth, Karlsruhe, Germany) in PBS or TBS (100 mM Tris, 150 mM NaCl in H_2O adjusted to pH 8.0) on a shaker at room temperature and incubated over night at 4°C with a primary antibody. Primary antibodies for c-MYC (RRID:AB_2151827, #9402S, Cell Signaling Technology, Danvers, Massachusetts, USA), PRMT5 (RRID:AB_2799945, #79998S, Cell Signaling Technology, Danvers, Massachusetts, USA), cleaved PARP (#51-9000017, BD Pharmingen, San Diego, California, USA), AURKB (RRID-Nr.: AB_10695307, #3094S, Cell Signaling Technology, Danvers, Massachusetts, USA), γH2Ax (RRID:AB_2755003, #05-636, Merck Millipore, Burlington, Massachusetts, USA), H4R3me2sy (#A-3718-050, EpiGentek Group Inc., Farmingdale, New York, USA), TPX2 (RRID:AB_10002747 Novus Biologicals, CO, USA, #NB500-179), E-Cadherin (RRID: AB_2291471, #3195, Cell Signaling Technology, Danvers, Massachusetts, USA), Vimentin (RRID: AB_10695459, #5741, Cell Signaling Technology, Danvers, Massachusetts, USA), BRD4 (RRID: AB_2687578, #13440, Cell Signaling Technology, Danvers, Massachusetts, USA) and β-Actin (RRID:AB_476743, #A5316, Sigma-Aldrich, Darmstadt, Germany) were used in a 1:1000 dilution in 5% (w/v) skim milk in PBS (cleaved PARP, MYC, and PRMT5), 1:750 dilution in 5% (w/v) skim milk in PBS (AURKB), or 1:1000 dilution in 5% (w/v) skim milk in TBS (γH2Ax and H4R3me2sy). After overnight incubation, a corresponding secondary antibody (anti-mouse or anti-rabbit DyLight™ 680 (RRID:AB_10696895, #5470 or RRID:AB_10693812, #5366S, Cell Signaling Technology, Danvers, Massachusetts, USA) or anti-mouse or anti-rabbit DyLight™ 800 (RRID:AB_10693543, #5257 or RRID:AB_10697505, #5151S, Cell Signaling Technology, Danvers, Massachusetts, USA)) conjugated secondary antibodies (1:10000 dilution in 5% (w/v) skim milk in PBS or TBS) was incubated with the membranes for 1 hour at room temperature. Between the antibody incubation times the membranes were washed three times

5 minutes with 0.1% Tween® 20 (#9127.2, Carl Roth, Karlsruhe, Germany) in PBS or TBS, respectively. Western blots were visualized using an Odyssey® Infrared Imaging System (RRID:SCR_013430, Licor Biosciences, Bad Homburg, Germany). Protein bands were quantified using the Image Studio Lite Software (RRID:SCR_013715, Licor Biosciences, Bad Homburg, Germany). Protein expression values were normalized on expression of a housekeeping protein (β-Actin) and final expression values were calculated out of 3 biological replicates or one replicate (Fig. 4D and 4F).

Quantitative PCR

The Maxwell® 16 LEV simply RNA Purification Kit (Promega, Walldorf, Germany) was used according to the manufacturer's instructions to isolate total RNA. cDNA was synthesized using 10x TaqMan RT Buffer (#4486220, Thermo Fisher Scientific, Waltham, Massachusetts, USA), $MgCl_2$ (25mM) (Peqlab, VWR International , Llc., West Chester, Pennsylvania, USA), dNTP-Mix 2.5mM (#331550, Biozym Scientific GmbH, Hessisch Oldendorf, Germany), Random Hexamers (#11034731001, Hoffmann-La Roche, Basel, Switzerland), RNase Inhibitor (#N8080119, Thermo Fisher Scientific, Waltham, Massachusetts, USA), and Multiscribe Reverse Transcriptase (50 U/µl) (#4308228, Thermo Fisher Scientific, Waltham, Massachusetts, USA). The cDNA was subsequently diluted 1:5 and used for quantification. Quantitative expression of mRNA was determined using a real-time PCR analysis system (TaqMan, PE StepOnePlus, Real-Time PCR System; Applied Biosystems Inc., Carlsbad, CA, USA) with Promega GoTaq qPCR Master Mix (#A6101, Promega, Walldorf, Germany) as fluorescent DNA binding dye. Data analysis was carried out with StepOne software (Applied Biosystem; Thermo Fisher Scientific, Waltham, MA, USA) according to the $\Delta\Delta$Ct method. Each sample was pipetted in triplicates, H_2O was used as a quality control and *Gapdh* was used as a housekeeping gene. The values were normalized to the housekeeping gene and values over three biological replicates were calculated. The following primers were used: murine: *GAPDH* fwd 5´G G G T T C C T A T A A A T A C G G A C T G C 3´; *GAPDH* rv 5´ T A C G G C C A A A T C C G T T C A C A 3´; *PRMT5* fwd 5´ C T G T C T T C C A T C C G C G T T T C A 3´; *PRMT5* rev 5´ G C A G T A G G T C T G A T C G T G T C T G 3´; human: *GAPDH* fwd 5`A A

T C C C A T C A C C A T C T T C C A 3`; *GAPDH* rev 5`T G G A C T C C A C G A C G T A C T C A 3`; *MYC* fwd 5` T C A G A G T C T G G A T C A C C T T C T G C T 3`; *MYC* rev 5` TG C G T A G T T G T G C T G A T G T G T G G A 3`; *PRMT5* fwd 5` G G C C A A G C A G G G G T T T G A T 3`; *PRMT5* rev 5` C A G T C C C T T C C T G A C A G C A G 3`

RNAseq

For RNA isolation, 100µl of RNA Solution supplemented with thioglycerol (#AS1280, Maxwell® 16 LEV simplyRNA Tissue Kit, Promega, Fitchburg, Wisconsin, USA) was pipetted onto the dishes, cells were collected using a cell scraper and frozen immediately at -80°C. RNA was isolated using the Maxwell® 16 LEV simplyRNA Tissue Kit and instrument (Promega, Fitchburg, Wisconsin, USA) following the manufacturers instructions. In the final step, RNA was eluted into RNase-free water and subsequently concentration was measured on a NanoDrop (Peqlab, VWR International, Llc., West Chester, Pennsylvania, USA).

RNA sequencing was performed at the Sequencing Core Unit at the TranslaTUM, Technical University Munich (TUM). Library preparation for bulk-sequencing of poly(A)-RNA was done as described previously (10). Briefly, barcoded cDNA of each sample was generated with a Maxima RT polymerase (Thermo Fisher) using oligo-dT primer containing barcodes, unique molecular identifiers (UMIs) and an adaptor. Ends of the cDNAs were extended by a template switch oligo (TSO) and full-length cDNA was amplified with primers binding to the TSO-site and the adaptor. NEB UltraII FS kit was used to fragment cDNA. After end repair and A-tailing a TruSeq adapter was ligated and 3'-end-fragments were finally amplified using primers with Illumina P5 and P7 overhangs. In comparison to Parekh et al. (10), the P5 and P7 sites were exchanged to allow sequencing of the cDNA in read1 and barcodes and UMIs in read2 to achieve a better cluster recognition. The library was sequenced on a NextSeq 500 (Illumina) with 67 cycles for the cDNA in read1 and 16 cycles for the barcodes and UMIs in read2. Data was processed using the published Drop-seq pipeline (v1.0) to generate sample- and gene-wise UMI tables (11). Reference genome (GRCm38 or GRCh38) was used for alignment. Transcript and gene definitions were used according to the GENCODE Version M25. RNAseq analysis was performed with R-Studio (R version 4.0.2 (2020-06-22), open source license) and

DEseq2 v 1.33.5 (12). Genes with sum (read counts) < 5 were removed and remaining counts were normalized and transformed using regularized log transformation (rlog) implemented in the DEseq2 package. The rlog normalized matrix was used to perform a GSEA using the GeneTrail 3.0 web tool with default setting. For finding of pathways associated with sensitive and resistant PDOs and PDCL, JNJ-64619178 GI_{50} values were quartalized and differential expressed genes (a) most sensitive versus least sensitive and b) most sensitive versus all other lines) were calculated using the DEseq2 package. Briefly, genes with sum (read counts) < n (number of Samples) were removed after collapsing replicates and remaining counts were used to calculate differential expressied genes using LFC-shrink and apeglm (13).

The log fold change was used as a rank to perform a pre-ranked GSEA via the GeneTrail 3.0 web tool. Expression datasets for JNJ-64619178 treated PSN1 and DanG cells and HPAC-MYC-CRISPRa can be accessed via ENA: PRJEB43040.

CRISPR Activation (CRISPRa) / CRISPR Interference (CRISPRi)

sgRNA cloning:

Following oligonucleotides specific for the target sequence were annealed: sgRNA-Myc2: fwd-Primer 5`C A C C G T T C C C C C A C G C C C T C T G C T T 3´; rev-Primer 5´ A A A C A A G C A G A G G G C G T G G G G G A A C 3´; sgRNA-PRMT5: fwd-Primer 5´ C A C C G A G C C G C G T G T C C A G C G G G A 3´; rev-Primer 5´ A A A C T C C C G C T G G A C A C G C G G C T C 3´. Annealing was performed adding 1 µl of the forward and reverse Primer (100 µM), 1 µl 10x T4 DNA Ligase Buffer (New England Biolabs, #B0202S) and 7 ul H_2O together in a 1.5 ml Eppendorf Tube. The solution was heated for 5 minutes at 95°C on a heating block, then removed and slowly cooled down for 30 minutes at room temperature. A 1:50 dilution was used for subsequent cloning. The PRMT5-CRISPRi-oligos were cloned into the lentiGuide-Puro vector (RRID:Addgene_52963)(14) and the MYC-CRISPRa-oligos into the vector lenti sgRNA(MS2)_zeo backbone (RRID:Addgene_61427) (15), by the by adding the following components: 1 µl vector (90 ng/µl), 1 µl annealed & diluted oligonucleotide, 2 µl 10x T4 DNA-Ligase Buffer (New England Biolabs, #B0202S), 1 µl T4 DNA Ligase (New England Biolabs, #M0202S), 1 µl BsmBI (New England Biolabs, #R0580S), and 14 µl H_2O. Mixture was

incubated in thermocycler with the following program: 10 cycles of (37°C for 5 minutes, 16°C for 10 minutes), 55°C for 5 minutes, 80°C for 5 minutes. Vectors were transformed in *E.coli Stbl3* bacteria and selected on LB-Agar (ROTH, #X969.2) plate with 100 µg/ml Ampicillin. Single colonies were amplified in LB-Medium supplemented with 100 µg/ml of Ampicillin. The plasmid was isolated with the "NucleoSpin Plasmid, Mini Kit for Plasmid DNA" (Machery Nagel, #740588.250) according to the manufactures protocol. All plasmids were sanger sequenced to test proper insertion and submitted to Addgene: sgRNA(MS2)_zeo insert c-MYC (Plasmid #164636) and lentiGuide-Puro PRMT5-CRISPRi (Plasmid #164637).

<u>Lentivirus cell production:</u>

1x10^6 HEK293FT cells (RRID:CVCL_6911) were seeded in a 10 cm dish in DMEM + 10% FBS. The next day, the medium was aspirated, and fresh medium was added. In addition, the transfection mix (18 µl Lipofectamin, 280 µl Optimem and 10 µl of a Plasmid mix (1.25µg psPAX2 (RRID:Addgene_12260) (psPAX2 was a gift from Didier Trono (EPFL, Lausanne, Switzerland)), 0.75 µg pMD2 (RRID:Addgene_12259) (pMD2 was a gift from Didier Trono), 2 µg of the different lenti-vectors filled to 10 µl with H_2O) was mixed and incubated for 5 minutes at room temperature added dropwise to the medium. On the following day, the medium was aspirated and new DMEM + 30% FBS was added. The same was done on the next day, but the aspirated medium was saved in a 15ml Falcon tube. On the last day, the medium was again aspirated and pooled with the medium of the previous day, filtered, and the lentivirus stored at -80°C.

<u>Lentiviral Cell transduction:</u>

100,000 – 150,000 cells were seeded in two wells of a 6-well plate for 24 hours. Afterwards, 1 ml of the lentivirus containing medium was added together with 8 µg/ml Polybrene. After 8 hours, 1 ml of Medium with 10% FBS was added and cultured overnight. Medium was refreshed and after additional 24 hours and cells were selected as follows: plentiGuide-puro selection with 2 µg/ml Puromycin (Invivogen, #ant-pr-1), sgRNA(MS2)_zeo backbone selection with 300 µg/ml zeocin (Invivogen, #ant-zn-1), plenti_dCas9-KRAB-MeCP2 selection with 400 µg/ml Geneticin (ThermoFisher scientific, # 10131035), plenti_dCAS-VP64_Blast

selection with 400 µg/ml Geneticin (ThermoFisher scientific, # 10131035), and plenti_MS2-P65-HSF1_Hygro selection with 800 µg/ml Hygromycin B (ThermoFisher scientific, # 10687010). The cell line Patu8988T PRMT5-CRISPRi were transduced with plenti_dCas9-KRAB-MeCP2 (RRID:Addgene_122205) and plentiGuide-Puro-sgRNA-PRMT5 (Plasmid #164637). The cell line HPAC-MYC-CRISPRa were transduced with plenti-dCAS-VP64_Blast (RRID:Addgene_61425), plenti_MS2-P65-HSF1_Hygro (RRID:Addgene_61426) and plenti_sgRNA(MS2)_zeo_sgRNA-MYC (Plasmid #164636).

Seahorse Analysis

1,000 – 2,000 cells per well were seeded in 80 µl of the respective cell line medium with 10% FCS in quadruplets in a Seahorse Cell Culture Plate. The wells at the edges were left empty. Parallelly, a 96 well plate was prepared the same way and all steps were performed identically to measure Hoechst staining for normalization. The next day, 20 nM JNJ-64619178 or DMSO for the control were added in 20 µl of medium. The day before the measurement, the cartridge was hydrated by adding 200 ul H_2O to each well. Furthermore, the RPMI and DMEM (#103576-100 and #102353-100 Agilent Technologies, USA) Seahorse medium was prepared by adding 2 mM Glutamine and 1 mM Pyruvate and adjusting the pH to 7.4. For the Mito Stress test, 25 mM D-glucose was added in addition to the others. The cartridge and calibration solution were incubated over night at 37°C and 0% CO_2.

The measurement was performed 72 hours after adding the drugs. One hour before the measurement, the medium in the seahorse plate was changed to 180 µl Seahorse medium and the H_2O in the cartridge was changed to 200 µl of prewarmed calibration solution. Both plates were incubated for 1 hour at 37°C and 0% CO_2. The ports in the Seahorse plate were loaded as followed. For the Mito Stress test: Port A) 20 µl of 20 µg/ml Oligomycin; Port B) 22 µl of 10 uM FCCP and 50 mM Pyruvate; Port C) 25 µl of 25 µM Rotenone and 25 µM AntimycinA. The port loading for the Glycolytic stress test was as followed: Port A) 20 µl of 100 mM Glucose; Port B) 22 µl of 20 ug/ml Oligomycin; Port C) 25 µl of 1 mM 2-desoxy-D-glucose. The plate was then analyzed in a Seahorse XFe96 Analyzer. The parallelly prepared 96-well plate was treated with 25 µl of 25 uM Hoechst-Staining and measured on a FLUOstar OPTIMA

microplate reader with a gain of 1500 (BMG Labtech GmbH, Ortenberg, Germany). The ECAR and OCR was calculated in relation to the plate with Hoechst-Staining in order to compensate for cell density. Calculation of the different parameters was done as described by the manufacturer (download available at http://www.agilent.com).

Glucose uptake assay

1,000 – 2,000 cells per well were seeded in 100 µl of the respective cell line medium with 10% FCS in white, clear-bottom 96-well plates (#3610, Corning Life Sciences, New York, USA). 20 nM of JNJ-64619178 were added in 20µl medium the next day. After 72 hours of treatment, glucose uptake was measured according to the Glucose Uptake Glo assay from Promega. Briefly, the 2-deoxyglucose-6-phosphate (2DG6P) reagent is made by mixing 100 µl luciferase reagent, 1 µl $NADP^+$ (20mM), 2.5 µl Glucose-6-Phosphate Dehydrogenase (G6PDH), 0.5 µl reductase and 0.0625 µl reductase substrate. The 2DG6P reagent is then incubated for 1 hour at room temperature. In the incubation time, the medium from the wells was aspirated and the cells were washed with 100 µl PBS. 50 µl of 1 mM 2-deoxyglucose (2DG) was added to each well, briefly shaken, and incubated for 10 minutes. As a next step, 25 µl Stop buffer were added and briefly shaken, before 25 µl of Neutralization buffer were added. As a last step, 100 µl of the prepared 2DG6P reagent is added to each well. Glucose uptake was measured after 1 hour incubation at room temperature on a FLUOstar OPTIMA microplate reader with a gain of 1500 (BMG Labtech GmbH, Ortenberg, Germany).

References

1. Burstin J von, et al. E-cadherin regulates metastasis of pancreatic cancer in vivo and is suppressed by a SNAIL/HDAC1/HDAC2 repressor complex. *Gastroenterology.* 2009;137(1):361–71, 371.e1–5.

2. Biederstädt A, et al. SUMO pathway inhibition targets an aggressive pancreatic cancer subtype. *Gut.* 2020;69(8):1472-1482.

3. Lankes K, et al. Targeting the ubiquitin-proteasome system in a pancreatic cancer subtype with hyperactive MYC. *Mol Oncol.* 2020;14(12):3048-3064.

4. Müller T, et al. Automated sample preparation with SP3 for low-input clinical proteomics. *Mol Syst Biol.* 2020;16(1):e9111.

5. Bian Y, et al. Robust, reproducible and quantitative analysis of thousands of proteomes by micro-flow LC–MS/MS. *Nat Commun.* 2020;11(1):157.

6. Xie Z, et al. Gene Set Knowledge Discovery with Enrichr. *Curr Protoc.* 2021;1(3):e90.

7. Conradt L, et al. Disclosure of Erlotinib as a Multikinase Inhibitor in Pancreatic Ductal Adenocarcinoma. *Neoplasia.* 2011;13(11):1026-IN24.

8. Ianevski A, Giri AK, Aittokallio T. SynergyFinder 2.0: visual analytics of multi-drug combination synergies. *Nucleic Acids Res.* 2020;48(W1):W488–W493.

9. Yadav B, et al. Searching for Drug Synergy in Complex Dose–Response Landscapes Using an Interaction Potency Model. *Comput Struct Biotechnology J.* 2015;13:504–513.

10. Parekh S, et al. The impact of amplification on differential expression analyses by RNA-seq. *Sci Rep-uk.* 2016;6(1):25533.

11. Macosko EZ, et al. Highly Parallel Genome-wide Expression Profiling of Individual Cells Using Nanoliter Droplets. *Cell.* 2015;161(5):1202–1214.

12. Love MI, Huber W, Anders S. Moderated estimation of fold change and dispersion for RNA-seq data with DESeq2. *Genome Biol.* 2014;15(12):550.

13. Zhu A, Ibrahim JG, Love MI. Heavy-tailed prior distributions for sequence count data: removing the noise and preserving large differences. *Bioinformatics.* 2019;35(12):2084–2092.

14. Sanjana NE, Shalem O, Zhang F. Improved vectors and genome-wide libraries for CRISPR screening. *Nat Methods.* 2014;11(8):783–784.

15. Konermann S, et al. Genome-scale transcriptional activation by an engineered CRISPR-Cas9 complex. *Nature.* 2015;517(7536):583–588.
