# Supplementary material for: Epigenetic drug screening defines a PRMT5 inhibitor–sensitive pancreatic cancer subtype
Source: JCI Insight. 2022 May 23;7(10):e151353. doi: 10.1172/jci.insight.151353 (PMC9220834; doi:10.1172/jci.insight.151353)
Supplement: Supplemental table 2 [file jciinsight-7-151353-s103.pdf]

Drug	Mode of Action<br>Target	Development
Gemcitabine	deoxycytidine analogue<br>RNR	clinical
Mitomycin C	intercalator	clinical
XL019	JAK2	clinical
Givinostat	HDAC	clinical
GSK591	PRMT5	preclinical
Resminostat	HDAC	clinical
CUDC-907	Dual PI3K / HDAC	clinical
M344	HDAC	preclinical
CUDC-101	Dual EGFR / HDAC	clinical
AZD1208	PIM	clinical
PCI-34051	HDAC	preclinical
AR-42	HDAC	clinical

Supplemental Tabel 2 *Targets and Developmental Stage*
